# Ambient temperature variability and newly diagnosed tuberculosis: Evidence from a time-stratified case-crossover study

**DOI:** 10.1097/EE9.0000000000000531

**Published:** 2026-10-06

**Authors:** Jingjing Zhang, Kun Xiang, Yongzhong Zhang, Qinghua Xu, Xiaohong Kan

**Affiliations:** a Anhui Provincial Center for Disease Control and Prevention, Hefei, China; b College & Hospital of Stomatology, Anhui Medical University, Anhui Provincial Key Laboratory of Oral Diseases Research, Hefei, China

**Keywords:** tuberculosis, extreme temperature, diurnal temperature ranges, time-stratified case-crossover study

## Abstract

**Background:**

Environmental factors, particularly temperature-related exposures, are increasingly recognized as important determinants of tuberculosis (TB) disease progression and symptom manifestation. However, the evidence regarding the associations of heat exposure, cold exposure, and diurnal temperature range (DTR) with newly diagnosed TB remains limited and inconsistent.

**Methods:**

Data from Anhui Province between 1 January 2015 and 31 December 2024 were analyzed using a two-stage analytical framework based on a time-stratified case-crossover study design. City-specific associations between temperature and TB were pooled using a random-effects meta-analysis. Effect estimates were presented as relative risks (RRs) with corresponding confidence intervals (CIs).

**Results:**

A total of 243,577 newly diagnosed TB cases were reported during the study period. Compared with the median daily mean temperature, extreme heat exposure (95th percentile of daily mean temperature) was positively associated with newly diagnosed TB (RR = 1.218, 95% CI = 1.171, 1.267). In contrast, extreme cold exposure (fifth percentile of daily mean temperature) was negatively associated with newly diagnosed TB (RR = 0.837, 95% CI = 0.784, 0.893). Additionally, a greater DTR (95th percentile) was positively associated with newly diagnosed TB compared with the median DTR (RR = 1.320, 95% CI = 1.153, 1.512). These associations remained robust across multiple sensitivity analyses.

**Conclusion:**

Environmental temperature conditions are significantly associated with TB diagnosis. These findings suggest that temperature variability may influence the timing of TB diagnosis and highlight the importance of incorporating meteorological information into climate-sensitive TB surveillance and public health preparedness.

**What this study adds**

This study represents the first multicity investigation of the associations between extreme temperature exposure and newly diagnosed tuberculosis across 16 cities in Anhui Province, China, over a 10-year period involving 243,577 cases. Using a time-stratified case-crossover design, we assessed the effects of extreme temperature and diurnal temperature range while controlling for seasonal trends and time-invariant confounders. Extreme heat exposure and large diurnal temperature range were positively associated with newly diagnosed tuberculosis, whereas cold exposure showed an inverse association. These findings highlight the potential role of temperature variability on the timing of tuberculosis diagnosis and support the integration of meteorological information into climate-sensitive tuberculosis surveillance and public health planning.

## Introduction

Tuberculosis (TB) is one of the leading causes of death among respiratory infectious diseases and is regarded as a global public health emergency.^1^ The 2024 World Health Organization Global TB Report identifies China as one of the countries with the heaviest TB burdens worldwide, highlighting the persistent challenges in TB prevention and control.^2^ Although the global increase in TB incidence has slowed in the post–coronavirus disease 2019 (COVID-19) era, likely attributable to disruptions in transmission dynamics and enhanced public health interventions during the pandemic, TB continues to pose a threat to population health.

The occurrence of TB is the result of a complex interplay of factors, including pathogen characteristics, host immunity, socioeconomic status, and environmental exposure.^3,4^ In recent years, against the backdrop of rapid urbanization and drastic climate change, increasing attention has been directed toward environmental factors in disease. Accumulating epidemiological evidence suggests that exposure to ambient air pollutants, including fine particulate matter (PM_2.5_) and gaseous pollutants, is associated with TB incidence.^5^ Meteorological factors such as temperature, relative humidity, and wind speed are also considered to shape TB transmission dynamics by influencing both pathogen survival and host susceptibility.^6^ Ambient temperature and its variability have become significant environmental stressors that may impact human health.^7^

Multiple studies have explored the association between temperature-related factors and TB progression. High temperature may be indirectly associated with TB through alterations in the concentration and chemical composition of air pollutants or by inducing systemic inflammatory responses that impair respiratory defense mechanisms.^8,9^ Exposure to cold environments may trigger mucosal irritation, epithelial damage, and immune dysregulation, thereby increasing susceptibility to respiratory infections.^10^ However, the evidence regarding the association between temperature changes and TB risk remains unclear. A multicenter study suggested that low temperatures and low relative humidity may promote TB transmission.^11^ Wu et al’s^12^ time-series analysis indicated that decreases in ambient temperature were associated with reductions in outpatient visits for respiratory diseases. Notably, temperature may influence TB epidemiology via behavioral pathways. For instance, extremely cold environments may reduce outdoor activities and limit interpersonal contact, potentially decreasing transmission opportunities and delaying seeking medical attention. Conversely, high temperatures may reduce airflow and increase time spent in enclosed environments, thereby facilitating transmission.

To explore the complex association between temperature-related exposures and TB, our multi-city study employed a time-stratified case-crossover design coupled with a two-stage analytical framework. This approach enables the assessment of short-term associations while controlling for time-invariant confounders and capturing potential nonlinear and lagged effects. The study aims to provide evidence to inform climate-sensitive TB surveillance and public health planning from an environmental perspective.

## Methods

### Study area

Anhui Province is located in eastern China, with geographical coordinates approximately ranging from 29°41’ to 34°38’ N latitude and 114°54’ to 119°37’ E longitude (Figure 1). Anhui is geographically located at an important junction between the coastal and inland regions, and its terrain is characterized by plains, hills, and low mountains. Anhui has a monsoon-influenced transitional climate between the warm temperate and subtropical zones, with four distinct seasons. The annual mean temperature ranges from approximately 14 to 17 °C, and the average annual precipitation ranges from about 800 to 1600 mm, with higher precipitation in southern areas than in northern areas. As of the end of 2024, Anhui Province had a resident population of 61.23 million, and TB has consistently ranked among the leading legally notifiable infectious diseases in recent years.

**Figure 1.**
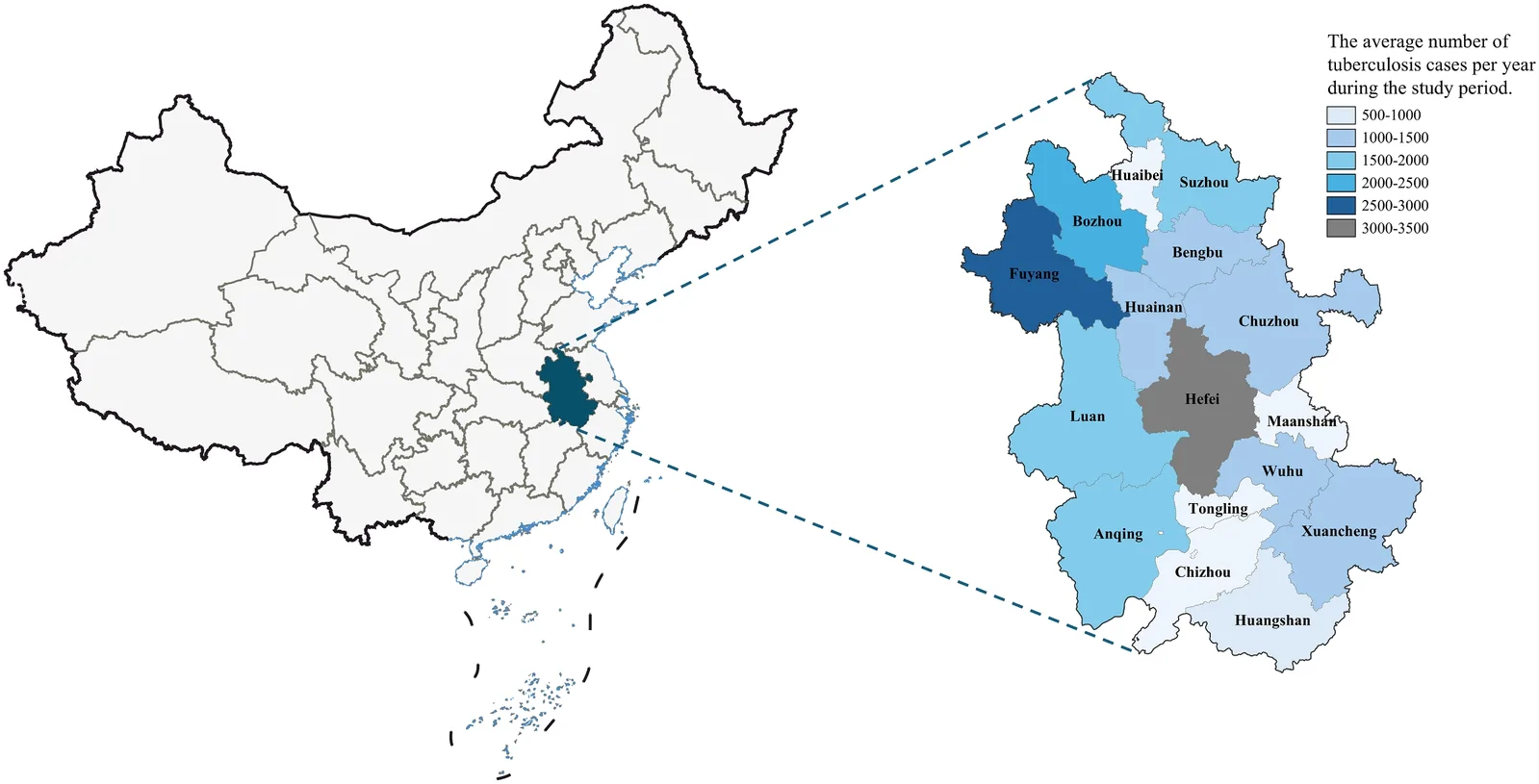
Geographical distribution of annual average newly diagnosed tuberculosis cases in sixteen cities of Anhui Province, 2015–2024.

### Study population

This study included a total of 243,577 newly diagnosed TB patients from 16 cities in Anhui Province during the period from 1 January 2015 to 31 December 2024. TB diagnoses were made in accordance with the most recent diagnostic criteria applicable in each year (WS 288-2008/WS 288-2017). As TB is classified as a legally notifiable infectious disease in China, healthcare institutions are required to report confirmed cases to the Chinese Center for Disease Control and Prevention online reporting system within 24 hours of diagnosis. TB data were obtained from the online reporting system of healthcare institutions, with information on patients’ age, sex, data of diagnosis, and place of residence extracted for analysis. To minimize potential bias associated with recurrent disease and enhance the reliability of the findings, this study only included newly diagnosed cases.

### Exposure assessment

Meteorological data were obtained from the ERA5-Land dataset developed by the European Centre for Medium-Range Weather Forecasts. ERA5-Land provides global land-surface meteorological variables at a spatial resolution of 0.1°×0.1° and hourly temporal resolution and has been widely used in environmental epidemiology to characterize ambient meteorological conditions. In the present study, daily meteorological variables extracted from the ERA5-Land grid cell corresponding to the administrative center of each prefecture-level city were used as proxies for city-level meteorological exposures. Hourly ambient temperature and relative humidity data were extracted and subsequently aggregated to derive daily meteorological indicators. Specifically, daily mean temperature was calculated as the average of hourly temperatures within a given day, while the daily minimum temperature and maximum temperature were defined as the lowest and highest hourly values recorded during the same period. Daily diurnal temperature range (DTR) was calculated as the difference between daily maximum and minimum temperatures. Daily relative humidity was calculated as the average of hourly values. Daily average concentrations of two air pollutants, including PM_2.5_ (μg/m^3^) and ozone (O_3_, μg/m^3^), were obtained from the National Urban Air Quality Real-time Release Platform (https://air.cnemc.cn:18007/). For each city, daily pollutant concentrations were calculated as the arithmetic mean of all available monitoring stations within the city. PM_2.5_ and O_3_ were included as covariates because they represent the major particulate and photochemical air pollutants, respectively, and have been associated with both ambient temperature and respiratory health outcomes.^13^

### Statistical analysis

A two-stage, multicity study was implemented to evaluate the associations between ambient temperature and newly diagnosed TB. In the first stage, the exposure–response relationship was estimated separately for each city. In the second stage, city-specific effect estimates were combined through meta-analysis to calculate an overall average effect across all study regions. In the first-stage analysis, a time-stratified case-crossover design was adopted to evaluate the association between ambient temperature variability and newly diagnosed TB. The analysis was conducted separately for each city using a conditional quasi-Poisson regression model combined with a Distributed Lag Nonlinear Model to simultaneously characterize nonlinear exposure–response relationships and delayed effects.^14,15^ Under the time-stratified framework, case and control days were matched by year, month, and day of the week, thereby constructing strata that inherently control for long-term trends, seasonality, and day-of-week effects. This design also eliminates confounding from individual characteristics that remain constant over the short observation window, such as age, sex, and socioeconomic status. Temperature effects were modeled using a cross-basis function within the Distributed Lag Nonlinear Model. The exposure–response dimension was modeled using a quadratic B-spline with three internal knots at the 25th, 50th, and 75th percentiles of the temperature distribution, while the lag-response dimension was modeled using a natural cubic spline to flexibly capture delayed effects of temperature exposure. This modeling strategy enables a flexible characterization of nonlinear exposure–response relationships and lagged effects of temperature on health outcomes. Based on previous studies related to respiratory diseases, this study specified a lag period of 0–21 days.^16,17^ Relative risks (RRs) and their 95% confidence intervals (CIs) were estimated by comparing the 5th and 95th percentiles of the temperature distribution with the median temperature, whereas for DTR, the comparison was made between the 95th percentile and the median DTR. In the second stage, region-specific estimates obtained in the first stage were pooled using a multivariate meta-analysis under a random-effects model to account for potential heterogeneity across study areas.^18^

To assess the potential interaction between temperature and air pollution in relation to newly diagnosed TB, we conducted a two-stage analysis. First, city-specific analyses were performed for each of the 16 cities. High temperature and high pollutant exposure were defined as daily values ≥75th percentiles, whereas low-temperature and low-pollutant exposure were defined as daily values ≤25th percentiles, based on city-specific distributions. Four joint-exposure scenarios were examined: high temperature with high PM_2.5_, high temperature with high O_3_, low temperature with high PM_2.5_, and low temperature with high O_3_. For each scenario, days with neither high temperature nor high air pollution were defined as the reference category. Daily TB counts were modeled using generalized linear models with a quasi-Poisson distribution and a log link. Additive interaction was assessed using the relative excess risk due to interaction and attributable proportion due to interaction (AP). Second, city-specific estimates were combined using random-effects meta-analysis with restricted maximum likelihood estimation.

All statistical tests were two-sided, with *P* < 0.05 considered statistically significant. All analyses were performed using R software (version 4.4.2).

### Subgroup analysis

To explore the differences in effects among different groups, we conducted stratified analysis based on multiple grouping variables. First, the above analytical procedures were repeated separately for males and females, as well as for different age groups (age <60 and age ≥60). In addition, potential effect modification by age and sex was assessed using heterogeneity tests based on *Z*-tests. Second, the study period was stratified into pre- and post-COVID-19 phases, using 31 January 2020,^19^ as the cutoff date, to assess whether COVID-19-related disruptions to healthcare services influenced TB diagnosis. Third, stratified analyses were conducted separately for the warm season (May to October) and the cold season (November to April) to assess potential seasonal heterogeneity in the observed associations. Fourth, given the substantial environmental differences between northern and southern Anhui Province, stratified analyses were conducted by geographic region (northern vs. southern) to assess potential regional heterogeneity. The stratified analysis facilitated the assessment of potential heterogeneity in the association between temperature and newly diagnosed TB among different populations, thereby assisting in identifying the groups that are more sensitive to temperature exposure and providing a direction for subsequent targeted research.

### Sensitivity analysis

To verify the robustness of the main research results, we further conducted sensitivity analyses. First, the primary analyses used a maximum lag of 21 days, while additional models with maximum lags of 14 and 28 days were fitted to examine the robustness of the results to different lag specifications. Second, considering the potential confounding effects of air pollution, sensitivity analyses were performed by incorporating PM_2.5_, O_3_, and relative humidity as covariates in additional models to account for potential co-exposure effects. Third, to assess the influence of model specification for the exposure–response relationship, alternative knot placements were tested for the temperature spline function. Specifically, the internal knots were shifted from the primary specification at the 25th, 50th, and 75th percentiles of the temperature distribution to alternative configurations at the 10th, 50th, and 90th percentiles and at the 10th, 75th, and 90th percentiles.^20^

## Results

### Descriptive results

A total of 243,577 newly diagnosed TB cases were included in this study between 1 January 2015 and 31 December 2024 (Table 1). Among these cases, 173,591 (71.3%) were male, and 69,986 (28.7%) were female. In terms of age distribution, 137,395 patients (56.4%) were younger than 60 years, while 106,182 (43.6%) were aged 60 years or older. During the study period, the mean ambient temperature in Anhui Province was 17.0 °C, with an average DTR of 7.89 °C. The mean concentrations of PM_2.5_ and O_3_ were 43.55 and 95.15 μg/m^3^, respectively, and the average relative humidity was 72.67%. These meteorological and air pollution variables were used to characterize environmental exposure conditions throughout the study period.

**Table 1.** Summary statistics of newly diagnosed TB cases, meteorological factors, and air pollutants in Anhui Province, 2015-2024

Variable	Number	Mean ± SD	Centiles				
Tuberculosis cases			Min	25%	50%	75%	Max
Total	243,577	4.17 ± 4.70	0	1	3	6	50
Anqing	19,784	5.42 ± 4.61	0	2	5	8	41
Bengbu	12,838	3.51 ± 3.60	0	1	3	5	30
Bozhou	21,119	5.78 ± 5.44	0	1	5	9	48
Chizhou	7,187	1.97 ± 2.27	0	0	1	3	28
Chuzhou	14,745	4.04 ± 4.02	0	1	3	6	36
Fuyang	29,443	8.06 ± 6.26	0	3	7	11	47
Hefei	33,075	9.05 ± 6.48	0	3	9	13	46
Huaibei	8,108	2.22 ± 2.30	0	0	2	3	18
Huainan	14,565	3.99 ± 4.03	0	0	3	6	28
Huangshan	5,763	1.58 ± 1.81	0	0	1	2	14
Luan	18,415	5.04 ± 4.97	0	1	4	8	48
Maanshan	8,834	2.42 ± 2.45	0	0	2	4	18
Suzhou	18,050	4.94 ± 5.37	0	0	4	7	50
Tongling	7,162	1.96 ± 2.43	0	0	1	3	26
Wuhu	12,428	3.40 ± 3.83	0	0	2	5	39
Xuancheng	12,061	3.30 ± 3.60	0	1	2	5	48
Gender							
Male	173,591	2.97 ± 3.47	0	0	2	4	42
Female	69,986	1.20 ± 1.69	0	0	1	2	23
Age							
<60	137,395	2.35 ± 2.93	0	0	1	3	34
≥60	106,182	1.82 ± 2.30	0	0	1	3	34
Temperature (°C)							
Mean temperature	/	17.00 ± 9.38	-8.62	8.86	17.80	25.17	35.01
DTR	/	7.89 ± 3.37	0.38	5.47	7.9	10.27	21.82
Pollutant concentration (μg/m^3^)							
PM_2.5_	/	43.55 ± 31.59	0	22	35	56	345
O_3_	/	95.15 ± 42.72	0	64	90	124	300
Relative humidity	/	72.67 ± 14.27	22.32	62.84	73.86	83.46	100

### Extreme temperature exposure results

Figure 2A illustrates the association between daily mean temperature and newly diagnosed TB. Figure 2B and C presents the lag–response relationships between extreme temperatures and newly diagnosed TB over lag 0–21 days. Compared with the median temperature, exposure to extremely high temperatures (95th percentile of daily mean temperature) was positively associated with TB diagnosis (RR = 1.171, 95% CI = 1.128, 1.216). In contrast, exposure to extremely low temperatures (fifth percentile) was negatively associated with TB diagnosis compared with the reference temperature (RR = 0.917, 95% CI = 0.869, 0.969). After adjusting for relative humidity and major air pollutants, the associations remained statistically significant (heat: RR = 1.218, 95% CI = 1.171, 1.267; cold: RR = 0.837, 95% CI = 0.784, 0.893). Table 2 summarizes the study findings on the relationship between extreme temperature exposure and newly diagnosed TB across 16 cities in Anhui Province. Supplementary Figure 1 https://links.lww.com/EE/A482 and Supplementary Figure 2 https://links.lww.com/EE/A483 illustrate the association between extreme temperature exposure and TB diagnosis across lag periods of 0–21 days in 16 cities of Anhui Province. No statistically significant synergistic interactions between temperature and air pollution were observed in relation to TB diagnosis. Neither high nor low temperature showed statistically significant additive interactions with PM_2.5_ or O_3_, with the estimated risk due to interaction and AP indicating no substantial departure from additivity (Supplementary Table 1 https://links.lww.com/EE/A470). Subgroup analyses yielded generally consistent results across sex and age groups, except for individuals aged <60 years under cold exposure, where the association was not statistically significant. Specifically, for high temperature exposure, the RRs were 1.161 (95% CI = 1.114, 1.211) for males, 1.198 (95% CI = 1.141, 1.257) for females, 1.200 (95% CI = 1.147, 1.255) for individuals aged <60 years and 1.135 (95% CI = 1.081, 1.191) for those aged ≥60 years. For low-temperature exposure, the RRs were 0.932 (95% CI = 0.882, 0.984) for males, 0.889 (95% CI = 0.830, 0.952) for females, 0.966 (95% CI = 0.912, 1.023) for individuals aged <60 years and 0.863 (95% CI = 0.811, 0.919) for those aged ≥60 years (Supplementary Table 2 https://links.lww.com/EE/A471). Significant heterogeneity was observed only for cold exposure across age groups. No statistically significant heterogeneity was observed between sex groups or age groups for the other exposure categories. In the stratified analysis by pandemic period, the associations observed during the pre-pandemic period were generally consistent with those identified in the main analysis, with the direction and magnitude of the associations showing similar patterns. In contrast, the associations observed during the post-pandemic period were attenuated and no longer reached statistical significance (Supplementary Table 3 https://links.lww.com/EE/A472). In the season-stratified analysis, the results for the cold season were generally consistent with those of the main analysis, whereas the association between extreme cold exposure and TB diagnosis was no longer statistically significant during the warm season (Supplementary Table 4 https://links.lww.com/EE/A473). Despite significant differences in geographical and environmental characteristics, as well as population behavioral patterns, between the southern and northern regions, the stratified analysis showed that the associations observed in both regions were broadly consistent with those identified in the main analysis (Supplementary Table 5 https://links.lww.com/EE/A474).

**Table 2.** Relative risks of newly diagnosed TB associated with extreme temperature exposure

City	Extremely temperature							
	Heat				Cold			
	RR^a^	95% CI	aRR^b^	95% CI	RR^a^	95% CI	aRR^b^	95% CI
Anqing	1.174	1.045, 1.319	1.230	1.093, 1.384	0.942	0.835, 1.062	0.848	0.743, 0.969
Bengbu	1.165	1.013, 1.339	1.273	1.100, 1.474	1.046	0.909, 1.204	0.912	0.775, 1.074
Bozhou	1.125	0.989, 1.280	1.124	0.975, 1.295	0.869	0.762, 0.992	0.901	0.775, 1.047
Chizhou	1.191	1.019, 1.392	1.206	1.029, 1.413	0.981	0.833, 1.155	0.943	0.781, 1.138
Chuzhou	1.127	0.987, 1.287	1.272	1.108, 1.461	0.928	0.807, 1.067	0.761	0.655, 0.885
Fuyang	1.067	0.958, 1.188	1.099	0.980, 1.233	0.973	0.874, 1.084	0.900	0.795, 1.019
Hefei	1.155	1.052, 1.269	1.193	1.082, 1.316	0.816	0.738, 0.903	0.764	0.681, 0.858
Huaibei	1.091	0.948, 1.256	1.061	0.913, 1.233	0.848	0.733, 0.981	0.864	0.726, 1.028
Huainan	1.050	0.918, 1.202	1.074	0.931, 1.239	0.822	0.714, 0.947	0.762	0.646, 0.899
Huangshan	1.239	1.064, 1.444	1.253	1.072, 1.466	0.837	0.701, 0.999	0.742	0.614, 0.898
Luan	1.181	1.036, 1.347	1.229	1.074, 1.406	0.735	0.638, 0.848	0.664	0.567, 0.777
Maanshan	1.058	0.922, 1.213	1.103	0.957, 1.272	0.857	0.742, 0.991	0.750	0.634, 0.888
Suzhou	0.978	0.840, 1.138	1.069	0.908, 1.257	0.829	0.712, 0.966	0.704	0.597, 0.829
Tongling	1.214	1.024, 1.438	1.274	1.070, 1.518	0.940	0.785, 1.124	0.840	0.688, 1.026
Wuhu	0.928	0.791, 1.089	1.005	0.855, 1.182	0.750	0.636, 0.884	0.620	0.518, 0.743
Xuancheng	1.092	0.942, 1.267	1.119	0.963, 1.301	0.843	0.718, 0.988	0.834	0.701, 0.992
Meta-results	1.171	1.128, 1.216	1.218	1.171, 1.267	0.917	0.869, 0.969	0.837	0.784, 0.893

a Unadjusted model.

b The model adjusted for PM_2.5_ and O_3_.

**Figure 2.**
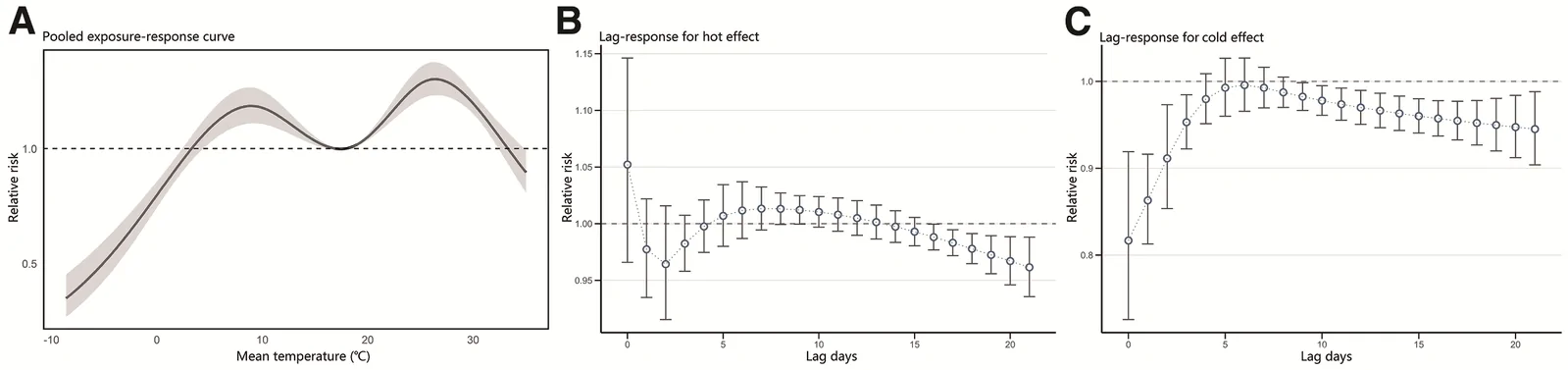
Exposure–response relationship between daily mean temperature and newly diagnosed tuberculosis (A), and lagged effects of extreme heat (B) and cold (C) on tuberculosis incidence (lag 0–21 days).

### Daily temperature range results

Figure 3 illustrates that the exposure–response curve exhibited a U-shaped relationship between DTR and newly diagnosed TB across a 21-day lag period. The results indicated that exposure to a greater DTR (95th percentile) was significantly associated with TB diagnosis (RR = 1.330, 95% CI = 1.170, 1.511). After adjusting for relative humidity and major air pollutants, the association remained statistically significant (RR = 1.320, 95% CI = 1.153, 1.512). Table 3 summarizes the associations between DTR and newly diagnosed TB across 16 cities in Anhui Province. Supplementary Figure 3 https://links.lww.com/EE/A484 and Supplementary Figure 4 https://links.lww.com/EE/A485 illustrate the association between extreme DTR and TB diagnosis in 16 cities in Anhui Province and their relationship over a lag period of 0–21 days. Subgroup analyses demonstrated statistically significant associations across both sex and age groups (male: RR = 1.351, 95% CI = 1.187, 1.538; female: RR = 1.304, 95% CI = 1.077, 1.578; age <60 years: RR = 1.279, 95% CI = 1.107, 1.478; age ≥60 years: RR = 1.401, 95% CI = 1.173, 1.673). No statistically significant heterogeneity was observed between sex groups or age groups (Supplementary Table 6 https://links.lww.com/EE/A475). In the analysis stratified by pandemic period, DTR was significantly associated with newly diagnosed TB during both the pre- and post-COVID-19 pandemic periods (Supplementary Table 7 https://links.lww.com/EE/A476). In the season-stratified analyses, DTR was significantly associated with newly diagnosed TB in both the cold and warm seasons (Supplementary Table 8 https://links.lww.com/EE/A477). In the subregional analyses, DTR was significantly associated with newly diagnosed TB in both northern and southern regions of the province (Supplementary Table 9 https://links.lww.com/EE/A478).

**Table 3.** Relative risks of newly diagnosed TB associated with DTR

City	DTR			
	RR^a^	95% CI	aRR^b^	95% CI
Anqing	0.954	0.675, 1.349	0.934	0.660, 1.322
Bengbu	1.278	0.838, 1.951	1.243	0.814, 1.897
Bozhou	1.871	1.299, 2.695	1.793	1.241, 2.591
Chizhou	1.541	0.915, 2.598	1.514	0.898, 2.553
Chuzhou	1.387	0.926, 2.078	1.431	0.952, 2.150
Fuyang	1.649	1.177, 2.310	1.694	1.205, 2.382
Hefei	1.218	0.902, 1.644	1.222	0.904, 1.651
Huaibei	1.226	0.795, 1.890	1.187	0.769, 1.833
Huainan	1.669	1.057, 2.637	1.670	1.057, 2.637
Huangshan	2.627	1.513, 4.563	2.557	1.469, 4.451
Luan	1.520	1.024, 2.256	1.499	1.010, 2.226
Maanshan	1.458	0.934, 2.277	1.504	0.961, 2.353
Suzhou	0.912	0.629, 1.321	0.904	0.623, 1.310
Tongling	1.493	0.818, 2.725	1.462	0.799, 2.675
Wuhu	0.981	0.575, 1.675	0.936	0.548, 1.600
Xuancheng	1.725	0.995, 2.990	1.775	1.023, 3.078
Meta-results	1.330	1.170, 1.511	1.320	1.153, 1.512

a Unadjusted model.

b The model adjusted for PM_2.5_ and O_3_.

CI indicates 95% confidence intervals; DTR, diurnal temperature range; RR, relative risk; TB, tuberculosis.

**Figure 3.**
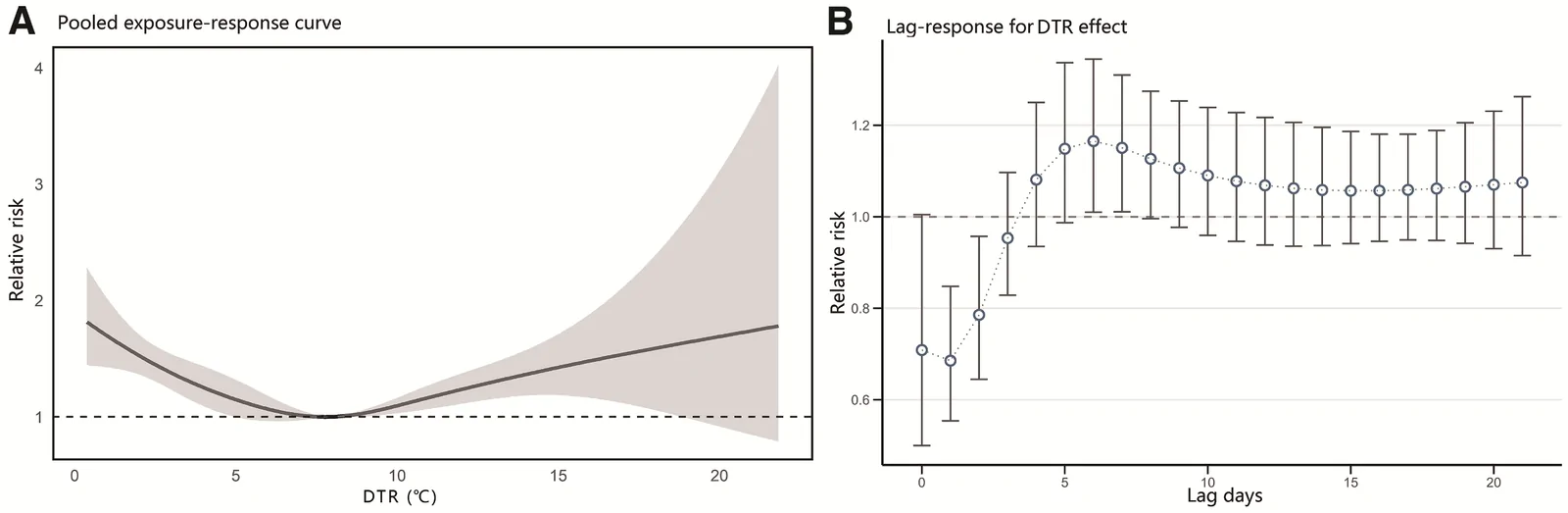
Exposure–response relationship between diurnal temperature ranges and newly diagnosed tuberculosis (A), and lagged effects of diurnal temperature ranges on tuberculosis incidence (lag 0–21 days) (B).

### Sensitivity analysis

After adjusting for major air pollutants and relative humidity in the crude model, the estimated associations remained largely unchanged, suggesting that the observed effects were not materially confounded by potential co-exposures. Furthermore, the results were consistent when varying the placement of knots in the spline functions used to model nonlinear exposure–response relationships (Supplementary Table 10 https://links.lww.com/EE/A479). These associations remained statistically significant when the maximum lag was extended to 28 days, with effect estimates broadly consistent with those observed in the primary analysis. When the maximum lag was shortened to 14 days, the associations for extreme temperature exposure remained statistically significant, whereas no clear association was observed between DTR and newly diagnosed TB (Supplementary Table 11 https://links.lww.com/EE/A480 and Supplementary Table 12 https://links.lww.com/EE/A481). Overall, the findings were broadly consistent across different model specifications, suggesting that the main findings were generally robust to alternative modeling specifications.

## Discussion

Using a time-stratified case-crossover design, this study is among the first to investigate the associations between temperature-related environmental exposures and newly diagnosed TB over a 10-year period in Anhui Province, China. The results suggested that extreme heat exposure and a greater DTR were positively associated with newly diagnosed TB, whereas extreme cold exposure showed an inverse association. These findings remained generally consistent across sensitivity analyses, supporting the robustness of the observed associations. Given the potentially prolonged interval between TB infection and diagnosis, our findings reflect associations with the timing of TB diagnosis rather than effects on TB transmission or incidence. Our results contribute to a growing body of evidence indicating that environmental and climatic conditions may be relevant to the temporal patterns of TB diagnosis.^21–23^

The observed positive association between heat exposure and TB diagnosis may be explained by a combination of behavioral, environmental, and biological mechanisms. Ambient temperature influences both pathogen viability and human social behavior, thereby jointly shaping the disease progression of airborne infectious diseases.^24^ Under extreme high-temperature conditions, individuals tend to stay in poorly ventilated enclosed indoor environments, potentially altering patterns of exposure to *Mycobacterium tuberculosis*. Inadequate indoor ventilation and high population density have been identified as important factors associated with TB.^25^ Furthermore, elevated ambient temperatures may influence host susceptibility. Previous studies have suggested that heat stress can disrupt immune homeostasis, impairing host defense mechanisms such as inflammatory responses and cellular immunity.^26^ These physiological changes may potentially alter host–pathogen interactions and could contribute to the observed association between extreme heat exposure and the timing of newly diagnosed TB.

Interestingly, our findings indicate a negative correlation between cold exposure and newly diagnosed TB. The result contrasts sharply with the seasonal peaks in TB notifications observed in many regions during winter or early spring.^1,27^ Extremely low temperatures may alter outdoor activities and social contact, which could partly account for the observed inverse association with the timing of newly diagnosed TB. Moreover, TB has a prolonged and variable interval between infection, symptom onset, and clinical detection, and the timing of diagnosis may not coincide with that of infection.^28^ Consequently, observed associations between environmental exposures and diagnosis may reflect complex temporal dynamics rather than direct causal effects.

The study also identified a significant positive correlation between DTR and TB diagnosis. Large diurnal temperature fluctuations may disrupt thermoregulatory homeostasis and impose physiological stress on the respiratory and immune systems.^9^ Such fluctuations may impair mucosal defense mechanisms, reducing the body’s resistance to respiratory pathogens. Previous studies have reported significant associations between DTR and the incidence and mortality rates of respiratory system diseases.^29,30^ Temperature variability may contribute to the reactivation of latent tuberculosis infection through modulation of immune function and increasing physiological stress. In summary, temperature fluctuations, rather than simply absolute temperature, may be relevant to the temporal patterns of TB diagnosis.

The subgroup analyses provide important insights into potential population heterogeneity in the association between temperature-related exposure and TB diagnosis. Given that young people are less affected by low temperatures, our results underscore the importance of accounting for age in the formulation of climate‑responsive TB control and monitoring plans.^31^ Following the onset of the COVID-19 pandemic, the association between extreme temperature exposure and TB diagnosis disappeared, providing new insights into how large-scale societal disruptions may modify the health effects of extreme heat. The seasonal heterogeneity observed in the subgroup analyses may partly reflect differences in temperature ranges and behavioral patterns across seasons. During the warm season, the relative rarity of extreme cold events, coupled with limited exposure contrast, may have reduced the statistical power to detect an association. Stratified analyses of northern and southern regions within the province suggested that the observed association may be relatively robust to regional environmental conditions and population behavioral patterns, despite substantial differences in geographic, environmental, and behavioral characteristics between the two regions. However, the association between DTR and TB diagnosis remained relatively consistent across subgroup analyses, highlighting the robustness of DTR as a stable meteorological risk factor for TB. In addition, the lag-response patterns indicated that the observed associations were concentrated primarily in the first few days after temperature exposure. This relatively short lag may reflect the influence of temperature on factors occurring close to the time of TB diagnosis, including short-term physiological responses, symptom manifestation, healthcare-seeking behavior, or other behavioral changes. However, given the prolonged and variable intervals from TB infection to diagnosis, the short-lag associations should be interpreted with caution, as they may reflect the temporal relationship between short-term temperature exposure and the timing of TB diagnosis rather than a direct impact on TB infection, transmission, or disease progression.

The frequency and intensity of extreme temperature events are projected to increase with global climate change, while amplifying temperature variability.^32^ Our findings are significant in the current context of climate change. Environmental changes may affect the temporal patterns of infectious diseases, including TB. Incorporating factors such as temperature and meteorology into TB surveillance systems may enhance early warning capabilities and facilitate the development of targeted prevention strategies. For instance, disease control systems could strengthen TB screening, health education, and community-based interventions during periods of extreme heat or large diurnal temperature variations. However, the existing evidence from East Asia regarding the association between temperature-related exposures and TB risk remains inconsistent. Several studies have reported a positive association between elevated or rapidly changing temperatures and TB risk. A study found that both greater temperature changes between neighboring days and higher DTR were significantly associated with an increased risk of TB hospitalization.^33^ Similarly, a study conducted in Japan reported that extreme heat exposure was associated with a 52.0% higher risk of TB incidence.^34^ Consistent with these findings, another study reported that increasing ambient temperature was associated with higher TB incidence and mortality, with each 1°C increase in annual mean temperature corresponding to 12.25% and 12.21% increases in the incidence and mortality rates of acute infectious TB, respectively.^35^ In contrast to the findings described above, a Chinese study using a generalized additive model reported that extreme high temperatures were associated with a reduced risk of TB.^36^ Moreover, contrary to our findings, several studies suggest that low temperatures are a potential risk factor for TB occurrence,^8,37^ whereas another study reported that extremely low temperatures were associated with a decreased incidence of TB.^38^ Taken together, the inconsistent findings across studies suggest that the association between temperature and TB is highly context-dependent and may vary according to local climatic conditions, population characteristics, exposure metrics, and study designs, underscoring the importance of accurately characterizing individual-level temperature exposure in epidemiological studies.

The study utilized a time-stratified case-crossover design to enable each case to serve as its own control, thereby effectively controlling for time-invariant confounding such as sex, genetics, and baseline socioeconomic characteristics. Importantly, the analysis used a long-term dataset spanning a decade, providing substantial statistical power and enabling the examination of long-term environmental influences on TB risk. At the same time, our study assessed multiple temperature-related exposure indicators simultaneously, allowing for a more comprehensive assessment of the effects of temperature variability on TB risk. The robustness of the findings was further validated by sensitivity analyses. Nevertheless, several limitations should be considered when interpreting the results. First, this study uses ERA5-Land meteorological data assigned to the grids containing the administrative centers of each city to estimate temperature exposure, rather than data based on individual residences. This approach may not adequately reflect the spatial variability of temperature within cities or at the individual level. Second, although the case-crossover design controls for many time-invariant confounding factors, residual confounding from unmeasured environmental or socioeconomic factors, such as housing density and nutritional status, may still exist. Third, the course of TB involves complex processes including infection, incubation period, and symptom onset. Therefore, the association found in this study should be interpreted as a relationship between environmental exposure and TB diagnosis, rather than as evidence of a direct impact on TB infection or disease onset. Fourth, the study used monitoring data from Anhui Province, so the results may not be directly generalizable to other regions with different climates or healthcare systems. Future research needs to incorporate multiregional datasets, individual-level exposure measurements, and additional environmental factors to clarify the mechanisms linking temperature variability and TB epidemiology.

## Conclusion

In summary, our study provides evidence for the association between temperature-related environmental factors and TB. High-temperature exposure and diurnal temperature variation were positively associated with newly diagnosed TB, while cold exposure was negatively correlated. These results highlight the potential role of climate variability in shaping temporal patterns of TB diagnosis and underscore the importance of incorporating environmental factors into TB surveillance, prevention, and control strategies. Given the ongoing impact of climate change on global temperature patterns, elucidating the interactions and mechanisms between environmental conditions and infectious diseases is crucial for effective public health planning and disease control strategies.

## Supplementary Material
