# Supplementary material for: Ambient temperature variability and newly diagnosed tuberculosis: Evidence from a time-stratified case-crossover study
Source: Environ Epidemiol. 2026 Oct 6;10(5):e531. doi: 10.1097/EE9.0000000000000531 (PMC13645473; doi:10.1097/EE9.0000000000000531)
Supplement: Supplementary file 1 [file ee9-10-e531-s001.pdf]

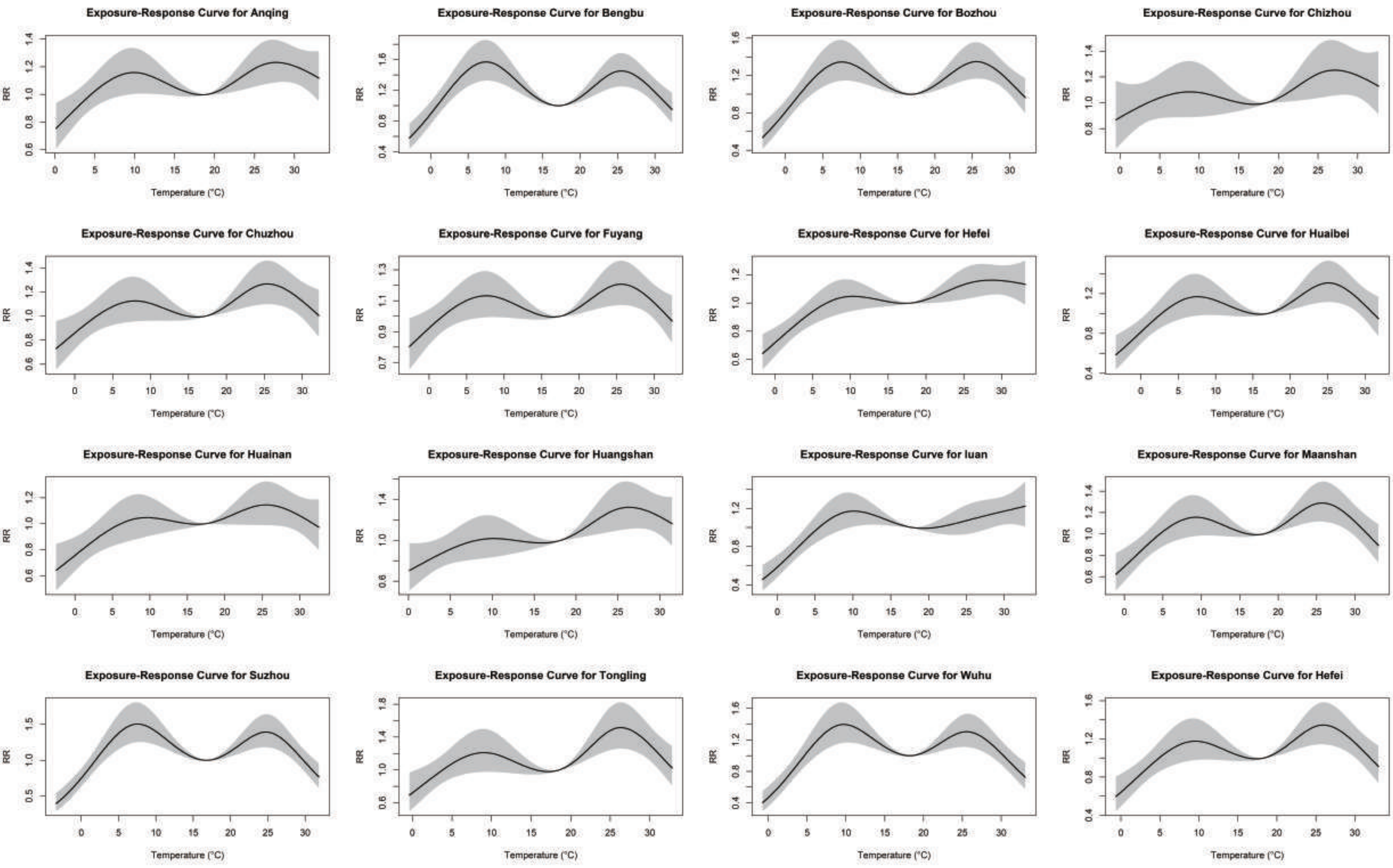
Exposure-Response Curve for Anqing
Exposure-Response Curve for Bengbu
Exposure-Response Curve for Bozhou
Exposure-Response Curve for Chizhou
Exposure-Response Curve for Chuzhou
Exposure-Response Curve for Fuyang
Exposure-Response Curve for Hefei
Exposure-Response Curve for Huaibei
Exposure-Response Curve for Huainan
Exposure-Response Curve for Huangshan
Exposure-Response Curve for luan
Exposure-Response Curve for Maanshan
Exposure-Response Curve for Suzhou
Exposure-Response Curve for Tongling
Exposure-Response Curve for Wuhu
Exposure-Response Curve for Hefei
RR
Temperature (°C)
