# Supplementary material for: Ambient temperature variability and newly diagnosed tuberculosis: Evidence from a time-stratified case-crossover study
Source: Environ Epidemiol. 2026 Oct 6;10(5):e531. doi: 10.1097/EE9.0000000000000531 (PMC13645473; doi:10.1097/EE9.0000000000000531)
Supplement: Supplementary file 2 [file ee9-10-e531-s002.pdf]

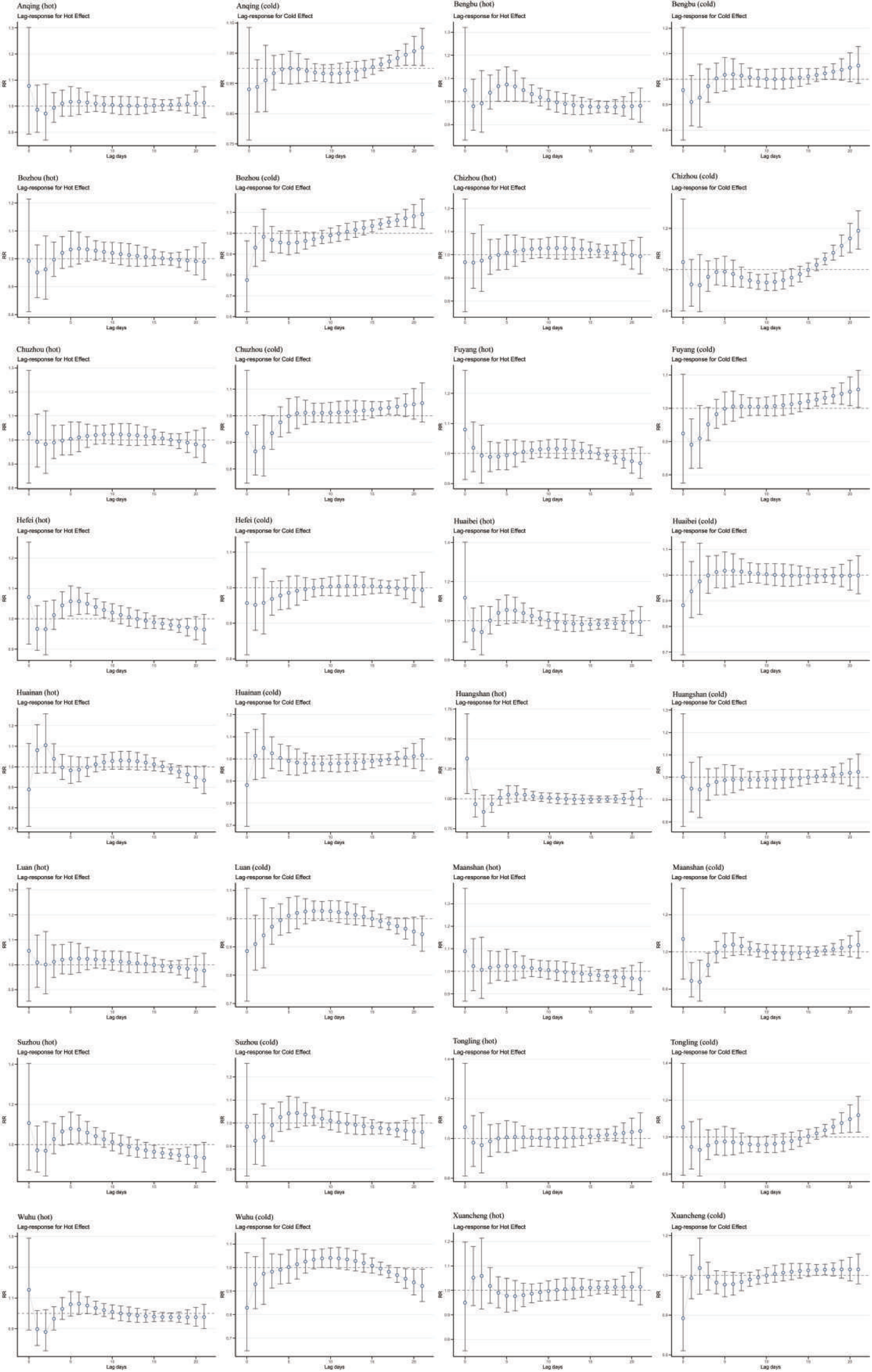
Anqing (hot)
Lag-response for Hot Effect
Anqing (cold)
Lag-response for Cold Effect
Bengbu (hot)
Lag-response for Hot Effect
Bengbu (cold)
Lag-response for Cold Effect
Bozhou (hot)
Lag-response for Hot Effect
Bozhou (cold)
Lag-response for Cold Effect
Chizhou (hot)
Lag-response for Hot Effect
Chizhou (cold)
Lag-response for Cold Effect
Chuzhou (hot)
Lag-response for Hot Effect
Chuzhou (cold)
Lag-response for Cold Effect
Fuyang (hot)
Lag-response for Hot Effect
Fuyang (cold)
Lag-response for Cold Effect
Hefei (hot)
Lag-response for Hot Effect
Hefei (cold)
Lag-response for Cold Effect
Huaibei (hot)
Lag-response for Hot Effect
Huaibei (cold)
Lag-response for Cold Effect
Huainan (hot)
Lag-response for Hot Effect
Huainan (cold)
Lag-response for Cold Effect
Huangshan (hot)
Lag-response for Hot Effect
Huangshan (cold)
Lag-response for Cold Effect
Luan (hot)
Lag-response for Hot Effect
Luan (cold)
Lag-response for Cold Effect
Maanshan (hot)
Lag-response for Hot Effect
Maanshan (cold)
Lag-response for Cold Effect
Suzhou (hot)
Lag-response for Hot Effect
Suzhou (cold)
Lag-response for Cold Effect
Tongling (hot)
Lag-response for Hot Effect
Tongling (cold)
Lag-response for Cold Effect
Wuhu (hot)
Lag-response for Hot Effect
Wuhu (cold)
Lag-response for Cold Effect
Xuancheng (hot)
Lag-response for Hot Effect
Xuancheng (cold)
Lag-response for Cold Effect
RR
Lag days
