# Supplementary material for: Ambient temperature variability and newly diagnosed tuberculosis: Evidence from a time-stratified case-crossover study
Source: Environ Epidemiol. 2026 Oct 6;10(5):e531. doi: 10.1097/EE9.0000000000000531 (PMC13645473; doi:10.1097/EE9.0000000000000531)
Supplement: Supplementary file 3 [file ee9-10-e531-s003.pdf]

Supplementary Table 1. Interaction Analysis Between $PM_{2.5}$ and O_3

City	Exposure	Multiplicative interaction		Additive interaction	
		RR (95% CI)	*P* for interaction	RERI	Attributable proportion
Anqing	High temp + High $PM_{2.5}$	1.179 (1.016 - 1.408)	0.165	0.133 (-0.039 - 0.376)	0.113 (-0.038 - 0.269)
	Low temp + High $PM_{2.5}$	1.047 (0.925 - 1.181)	0.426	-0.037 (-0.160 - 0.073)	-0.036 (-0.161 - 0.064)
	High temp + High O_3	1.034 (0.945 - 1.129)	0.482	-0.059 (-0.169 - 0.051)	-0.057 (-0.167 - 0.048)
	Low temp + High O_3	0.918 (0.672 - 1.171)	0.553	-0.131 (-0.417 - 0.110)	-0.143 (-0.608 - 0.095)
Bengbu	High temp + High $PM_{2.5}$	0.957 (0.709 - 1.212)	0.713	-0.128 (-0.400 - 0.163)	-0.134 (-0.535 - 0.131)
	Low temp + High $PM_{2.5}$	0.971 (0.864 -1.186)	0.682	-0.023 (-0.148 - 0.143)	-0.024 (-0.149 - 0.137)
	High temp + High O_3	1.011 (0.903 - 1.127)	0.850	-0.008 (-0.143 - 0.125)	-0.008 (-0.143 - 0.123)
	Low temp + High O_3	1.084 (0.575 - 2.560)	0.823	0.165 (-0.377 - 1.588)	0.152 (-0.653 - 0.624)
Bozhou	High temp + High $PM_{2.5}$	1.014 (0.779 - 1.243)	0.931	0.057 (-0.209 - 0.287)	0.057 (-0.253 - 0.228)
	Low temp + High $PM_{2.5}$	0.942 (0.818 - 1.103)	0.407	0.015 (-0.096 - 0.152)	0.016 (-0.099 - 1.151)
	High temp + High O_3	0.973 (0.883 - 1.071)	0.614	-0.036 (-0.154 - 0.092)	-0.037 (-0.165 - 0.091)
	Low temp + High O_3	0.716 (0.494 - 1.032)	0.245	-0.255 (-0.508 - 0.071)	-0.356 (-0.989 - 0.069)
Chizhou	High temp + High $PM_{2.5}$	0.817 (0.538 - 1.241)	0.230	-0.028 (-0.357 - 0.413)	-0.034 (-0.658 - 0.326)
	Low temp + High $PM_{2.5}$	1.063 (0.902 - 1.286)	0.498	0.002 ('-0.137 - 0.236)	0.021 (-0.134 - 0.210)
	High temp + High O_3	0.875 (0.766 - 1.026)	0.069	-0.012 (-0.155 - 0.143)	-0.014 (-0.184 - 0.148)
	Low temp + High O_3	0.738 (0.169 - 1.221)	0.538	-0.354 (-0.943 - 0.101)	-0.479 (-5.252 - 0.087)
Chuzhou	High temp + High $PM_{2.5}$	0.888 (0.709 - 1.064)	0.291	-0.069 (-0.235 - 0.136)	-0.077 (-0.320 - 0.128)
	Low temp + High $PM_{2.5}$	1.002 (0.880 - 1.139)	0.974	0.067 (-0.043 - 0.213)	0.067 (-0.045 - 0.211)
	High temp + High O_3	0.888 (0.770 - 0.989)	0.043	0.008 (-0.103 - 0.117)	0.009 (-0.118 - 0.133)
	Low temp + High O_3	0.884 (0.164 - 1.979)	0.718	-0.019 (-0.784 - 1.101)	-0.022 (-5.070 - 0.547)
Fuyang	High temp + High $PM_{2.5}$	0.914 (0.723 - 1.178)	0.535	-0.053 (-0.223 - 0.208)	-0.058 (-0.286 - 0.176)
	Low temp + High $PM_{2.5}$	0.937 (0.840 - 1.058)	0.266	-0.021 (-0.131 - 0.088)	-0.023 (-0.140 - 0.088)
	High temp + High O_3	0.997 (0.916 - 1.089)	0.951	-0.002 (-0.118 - 0.127)	-0.002 (-0.117 - 0.127)
	Low temp + High O_3	1.262 (0.811 - 1.823)	0.352	0.282 (-0.197 - 0.797)	0.224 (-0.252 - 0.449)
Hefei	High temp + High $PM_{2.5}$	0.923 (0.817 - 1.062)	0.321	-0.030 (-0.140 - 0.122)	-0.033 (-0.167 - 0.116)
	Low temp + High $PM_{2.5}$	0.981 (0.893 - 1.104)	0.715	-0.021 (-0.116 - 0.080)	-0.022 (-0.116 - 0.079)
	High temp + High O_3	0.962 (0.888 - 1.023)	0.316	-0.037 (-0.143 - 0.054)	-0.038 (-0.149 - 0.055)
	Low temp + High O_3	0.879 (0.001 - 1.298)	0.725	-0.129 (-1.033 - 0.255)	-0.147 (-171738.895 - 0.197)
Huaibei	High temp + High $PM_{2.5}$	0.932 (0.752 - 1.095)	0.654	-0.137 (-0.314 - 0.055)	-0.147 (-0.397 - 0.054)
	Low temp + High $PM_{2.5}$	0.990 (0.820 - 1.147)	0.898	0.038 (-0.113 - 0.214)	0.038 (-0.118 - 0.212)
	High temp + High O_3	0.982 (0.875 - 1.069)	0.761	-0.030 (-0.166 - 0.117)	-0.031 (-0.175 - 0.115)
	Low temp + High O_3^*	-	-	-	-
Huainan	High temp + High $PM_{2.5}$	0.595 (0.444 - 0.783)	0.036	-0.493 (-0.693 - -0.269)	-0.829 (-1.423 - -0.351)
	Low temp + High $PM_{2.5}$	1.107 (0.963 - 1.278)	0.264	-0.064 (-0.246 - 0.089)	-0.058 (-0.230 - 0.082)
	High temp + High O_3	0.912 (0.807 - 1.025)	0.187	-0.047 (-0.183 - 0.063)	-0.051 (-0.209 - 0.071)
	Low temp + High O_3	0.570 (0.001 - 0.934)	0.361	-0.388 (-0.898 - -0.012)	-0.681 (-175283.199 - -0.013)
Huangshan	High temp + High $PM_{2.5}$	0.116 (0.839 - 1.442)	0.401	0.144 (-0.188 - 0.485)	0.129 (-0.235 - 0.341)
	Low temp + High $PM_{2.5}$	1.047 (0.885 - 1.263)	0.622	-0.071 (-0.269 - 0.122)	-0.068 (-0.260 - 0.110)
	High temp + High O_3	0.930 (0.792 - 1.093)	0.396	-0.097 (-0.236 - 0.094)	-0.104 (-0.264 - 0.100)
	Low temp + High O_3	0.903 (0.686 - 1.182)	0.521	-0.129 (-0.398 - 0.174)	-0.143 (-0.531 - 0.152)
Luan	High temp + High $PM_{2.5}$	1.164 (0.900 - 1.416)	0.194	0.149 (-0.133 - 0.398)	0.128 (-0.136 - 0.289)
	Low temp + High $PM_{2.5}$	1.004 (0.878 - 1.131)	0.953	-0.040 (-0.165 - 0.092)	-0.040 (-0.173 - 0.089)
	High temp + High O_3	1.032 (0.916 - 1.148)	0.561	0.033 (-0.095 - 0.158)	0.032 (-0.090 - 0.153)
	Low temp + High O_3	1.099 (0.478 - 1.861)	0.748	0.093 (-0.514 - 0.810)	0.085 (-1.076 - 0.451)
Maanshan	High temp + High $PM_{2.5}$	0.868 (0.693 - 1.073)	0.212	-0.220 (-0.407 - -0.002)	-0.253 (-0.574 - -0.002)
	Low temp + High $PM_{2.5}$	0.981 (0.867 - 1.135)	0.799	0.051 (-0.050 - 0.197)	0.052 (-0.053 - 0.199)
	High temp + High O_3	0.981 (0.855 - 1.097)	0.738	0.005 (-0.140 - 0.140)	0.005 (-0.145 - 0.139)
	Low temp + High O_3	0.661 (0.529 - 0.827)	0.569	-0.240 (-0.421 - -0.068)	-0.363 (-0.750 - -0.084)
Suzhou	High temp + High $PM_{2.5}$	1.015 (0.831 - 1.232)	0.897	-0.045 (-0.232 - 0.166)	-0.044 (-0.270 - 0.147)
	Low temp + High $PM_{2.5}$	1.052 (0.906 - 1.213)	0.447	0.009 (-0.139 - 0.162)	0.008 (-0.132 - 0.150)
	High temp + High O_3	1.014 (0.912 - 1.127)	0.797	-0.046 (-0.173 - 0.074)	-0.045 (-0.165 - 0.073)
	Low temp + High O_3	0.897 (0.477 - 1.287)	0.602	-0.116 (-0.520 - 0.254)	-0.129 (-1.091 - 0.204)

Tongling	High temp + High $PM_{2.5}$	1.241 (0.957 - 1.533)	0.134	0.292 ('-0.009 - 0.595)	0.235 (-0.010 - 0.399)
	Low temp + High $PM_{2.5}$	1.291 (1.074 - 1.573)	0.014	-0.100 (-0.345 - 0.118)	-0.077 (-0.292 - 0.086)
	High temp + High O_3	0.820 (0.686 - 0.977)	0.014	-0.222 (-0.420 - -0.022)	-0.271 (-0.536 - -0.028)
	Low temp + High O_3	1.616 (0.001 - 2.934)	0.304	0.572 ('-1.030 - 1.844)	0.354 (-29397.373 - 0.648)
Wuhu	High temp + High $PM_{2.5}$	0.889 (0.663 - 1.154)	0.531	-0.173 (-0.404 - 0.109)	-0.194 (-0.602 - 0.103)
	Low temp + High $PM_{2.5}$	0.883 (0.756 - 1.079)	0.195	0.029 (-0.109 - 0.201)	0.033 (-0.118 - 0.211)
	High temp + High O_3	1.067 (0.912 - 1.231)	0.397	0.084 (-0.090 - 0.231)	0.079 (-0.092 - 0.214)
	Low temp + High O_3	1.774 (0.275 - 3.159)	0.101	0.864 (-0.597 - 2.217)	0.487 (-2.215 - 0.708)
Xuancheng	High temp + High $PM_{2.5}$	0.915 (0.768 - 1.134)	0.613	0.032 (-0.135 - 0.256)	0.035 (-0.172 - 0.243)
	Low temp + High $PM_{2.5}$	0.910 (0.788 - 1.109)	0.304	0.087 (-0.054 - 0.268)	0.095 (-0.056 - 0.277)
	High temp + High O_3	0.916 (0.793 - 1.050)	0.250	-0.177 (-0.328 - 0.013)	-0.193 (-0.371 - 0.013)
	Low temp + High O_3	1.103 (0.632 - 1.719)	0.552	0.136 (-0.376 - 0.657)	0.123 (-0.564 - 0.417)
Meta-results	High temp + High $PM_{2.5}$	0.964 (0.893 - 1.041)	0.349	-0.048 (-0.135 - 0.039)	-0.013 (-0.085 - 0.059)
	Low temp + High $PM_{2.5}$	1.003 (0.968 - 1.040)	0.867	-0.001 (-0.036 - 0.034)	-0.002 (-0.036 - 0.033)
	High temp + High O_3	0.973 (0.947 - 1.001)	0.051	-0.031 (-0.064 - 0.002)	-0.029 (-0.063 - 0.005)
	Low temp + High O_3	0.483 (0.125 - 1.875)	0.293	-0.176 (-0.362 - 0.010)	-0.081 (-0.243 - 0.082)

$PM_{2.5}$: fine particulate matter; O_3: ozone; RR: relative risk; CI: 95% confidence intervals; RERI: relative excess risk due to interaction.

*The number of days that meet both low temperature and high ozone standards is too few, making it impossible to conduct the analysis.
