# Supplementary material for: Ambient temperature variability and newly diagnosed tuberculosis: Evidence from a time-stratified case-crossover study
Source: Environ Epidemiol. 2026 Oct 6;10(5):e531. doi: 10.1097/EE9.0000000000000531 (PMC13645473; doi:10.1097/EE9.0000000000000531)
Supplement: Supplementary file 4 [file ee9-10-e531-s004.pdf]

Supplementary Table 2. Sex-stratified analysis of the association between extreme temperature exposure and newly diagnosed TB

City	Extremely heat exposure						Extremely cold exposure					
	Male	Female	*P* for heterogeneity	Age<60	Age⩾60	*P* for heterogeneity	Male	Female	*P* for heterogeneity	Age<60	Age⩾60	*P* for heterogeneity
Anqing	1.166 (1.033-1.317)	1.199 (1.027-1.401)	0.781	1.187 (1.035-1.361)	1.158 (1.017-1.320)	0.798	0.946 (0.835-1.071)	0.939 (0.800-1.103)	0.943	1.021 (0.890-1.172)	0.856 (0.746-0.981)	0.075
Bengbu	1.177 (1.017-1.363)	1.125 (0.929-1.363)	0.713	1.201 (1.032-1.397)	1.110 (0.932-1.321)	0.504	1.045 (0.920-1.210)	1.070 (0.883-1.297)	0.844	1.046 (0.898-1.219)	1.061 (0.893-1.261)	0.904
Bozhou	1.066 (0.930-1.222)	1.257 (1.074-1.470)	0.120	1.233 (1.068-1.423)	1.022 (0.884-1.182)	0.072	0.880 (0.767-1.009)	0.852 (0.723-1.005)	0.767	0.987 (0.854-1.142)	0.769 (0.663-0.892)	0.018
Chizhou	1.167 (0.983-1.385)	1.245 (0.997-1.554)	0.651	1.172 (0.973-1.143)	1.208 (1.004-1.454)	0.769	0.994 (0.832-1.188)	0.932 (0.734-1.185)	0.672	1.035 (0.855-1.253)	0.918 (0.752-1.121)	0.395
Chuzhou	1.115 (0.970-1.281)	1.161 (0.977-1.379)	0.720	1.191 (1.025-1.384)	1.058 (0.910-1.231)	0.276	0.942 (0.814-1.090)	0.883 (0.733-1.063)	0.592	0.980 (0.836-1.148)	0.870 (0.741-1.020)	0.300
Fuyang	1.048 (0.938-1.171)	1.121 (0.979-1.284)	0.451	1.128 (1.004-1.268)	0.988 (0.872-1.119)	0.128	0.956 (0.855-1.069)	1.021 (0.890-1.171)	0.466	1.031 (0.916-1.160)	0.897 (0.792-1.016)	0.112
Hefei	1.127 (1.020-1.246)	1.217 (1.087-1.364)	0.320	1.145 (1.034-1.268)	1.171 (1.049-1.306)	0.769	0.824 (0.740-0.916)	0.798 (0.704-0.904)	0.702	0.837 (0.751-0.934)	0.781 (0.693-0.880)	0.401
Huaibei	1.102 (0.945-1.287)	1.069 (0.878-1.300)	0.881	1.116 (0.951-1.309)	1.058 (0.877-1.276)	0.671	0.852 (0.726-0.999)	0.841 (0.687-1.031)	0.921	0.897 (0.761-1.057)	0.763 (0.627-0.929)	0.216
Huainan	1.035 (0.897-1.194)	1.089 (0.917-1.293)	0.656	1.018 (0.878-1.180)	1.094 (0.932-1.283)	0.517	0.828 (0.713-0.961)	0.801 (0.667-0.961)	0.783	0.830 (0.711-0.968)	0.808 (0.683-0.958)	0.818
Huangshan	1.287 (1.088-1.522)	1.108 (0.877-1.401)	0.380	1.237 (1.017-1.503)	1.229 (1.020-1.481)	0.962	0.897 (0.739-1.087)	0.696 (0.526-0.919)	0.143	0.912 (0.730-1.139)	0.762 (0.612-0.950)	0.260
Luan	1.161 (1.012-1.332)	1.232 (1.047-1.450)	0.580	1.156 (1.001-1.335)	1.209 (1.041-1.405)	0.683	0.710 (0.611-0.825)	0.805 (0.676-0.959)	0.286	0.797 (0.684-0.928)	0.674 (0.570-0.796)	0.146
Maanshan	1.067 (0.916-1.243)	1.046 (0.866-1.263)	0.872	1.111 (0.942-1.311)	0.998 (0.848-1.174)	0.365	0.896 (0.763-1.051)	0.770 (0.628-0.945)	0.253	0.888 (0.745-1.059)	0.820 (0.690-0.974)	0.526
Suzhou	0.940 (0.804-1.100)	1.091 (0.908-1.311)	0.227	1.010 (0.861-1.184)	0.937 (0.783-1.120)	0.539	0.800 (0.684-0.937)	0.918 (0.763-1.106)	0.268	0.879 (0.748-1.032)	0.769 (0.643-0.918)	0.275
Tongling	1.197 (0.998-1.436)	1.273 (1.009-1.606)	0.683	1.177 (0.970-1.428)	1.262 (1.031-1.544)	0.625	0.990 (0.818-1.199)	0.785 (0.605-1.018)	0.159	0.873 (0.710-1.074)	1.000 (0.806-1.242)	0.374
Wuhu	0.918 (0.776-1.087)	0.949 (0.774-1.162)	0.805	0.953 (0.796-1.140)	0.891 (0.738-1.074)	0.612	0.795 (0.670-0.943)	0.634 (0.511-0.788)	0.108	0.784 (0.653-0.941)	0.707 (0.583-0.858)	0.446
Xuancheng	1.104 (0.947-1.288)	1.072 (0.876-1.312)	0.820	1.024 (0.866-1.211)	1.160 (0.979-1.373)	0.305	0.867 (0.735-1.023)	0.774 (0.622-0.965)	0.418	0.820 (0.686-0.980)	0.863 (0.718-1.037)	0.696
Meta-results	1.161 (1.114-1.211)	1.198 (1.141-1.257)	0.336	1.200 (1.147-1.255)	1.135 (1.081-1.191)	0.099	0.932 (0.882-0.984)	0.889 (0.830-0.952)	0.291	0.966 (0.912-1.023)	0.863 (0.811-0.919)	0.009

TB: tuberculosis; RR: relative risk; CI: 95% confidence intervals.
