# Supplementary material for: Ambient temperature variability and newly diagnosed tuberculosis: Evidence from a time-stratified case-crossover study
Source: Environ Epidemiol. 2026 Oct 6;10(5):e531. doi: 10.1097/EE9.0000000000000531 (PMC13645473; doi:10.1097/EE9.0000000000000531)
Supplement: Supplementary file 5 [file ee9-10-e531-s005.pdf]

Supplementary Table 3. Stratified analysis of the associations between extreme temperature exposure and newly diagnosed TB before and after the COVID-19 pandemic

City	Pre-pandemic period								Post-pandemic period							
	Heat				Cold				Heat				Cold			
	RR*	95% CI	aRR**	95% CI	RR*	95% CI	aRR**	95% CI	RR*	95% CI	aRR**	95% CI	RR*	95% CI	aRR**	95% CI
Anqing	1.378	1.179 - 1.610	1.368	1.166 - 1.605	0.908	0.776 - 1.063	0.960	0.783 - 1.178	1.030	0.880 - 1.207	1.038	0.885 - 1.216	0.938	0.792 - 1.110	0.968	0.810 - 1.157
Bengbu	1.404	1.180 - 1.669	1.372	1.143 - 1.646	0.861	0.714 - 1.038	0.884	0.700 - 1.116	0.994	0.822 - 1.202	0.976	0.795 - 1.200	1.065	0.885 - 1.282	1.217	0.984 - 1.505
Bozhou	1.552	1.311 - 1.837	1.522	1.266 - 1.829	0.591	0.491 - 0.711	0.692	0.557 - 0.860	0.847	0.704 - 1.019	0.770	0.627 - 0.947	1.067	0.890 - 1.280	1.200	0.979 - 1.470
Chizhou	1.373	1.098 - 1.718	1.337	1.061 - 1.685	1.002	0.816 - 1.229	1.043	0.803 - 1.356	1.087	0.883 - 1.340	1.081	0.876 - 1.335	0.970	0.759 - 1.240	1.019	0.775 - 1.338
Chuzhou	1.361	1.146 - 1.618	1.389	1.156 - 1.668	0.759	0.627 - 0.918	0.710	0.560 - 0.900	0.931	0.776 - 1.116	0.948	0.781 - 1.150	1.001	0.830 - 1.209	0.978	0.802 - 1.194
Fuyang	1.253	1.090 - 1.442	1.205	1.036 - 1.401	0.941	0.826 - 1.072	1.033	0.877 - 1.218	0.988	0.857 - 1.138	0.939	0.807 - 1.092	0.938	0.805 - 1.093	0.992	0.838 - 1.176
Hefei	1.352	1.187 -1.541	1.358	1.180 - 1.562	0.799	0.698 - 0.914	0.761	0.641 - 0.902	1.022	0.896 - 1.164	1.024	0.894 - 1.174	0.846	0.730 - 0.981	0.852	0.724 - 1.003
Huaibei	1.403	1.168 - 1.685	1.315	1.085 - 1.594	0.739	0.605 - 0.902	0.704	0.548 - 0.903	0.868	0.702 - 1.073	0.924	0.731 - 1.167	0.893	0.729 - 1.093	1.007	0.795 - 1.275
Huainan	1.167	0.977 - 1.394	1.110	0.919 - 1.340	0.707	0.576 - 0.869	0.616	0.475 - 0.798	0.984	0.805 - 1.203	1.013	0.815 - 1.258	0.867	0.717 - 1.049	0.953	0.766 - 1.185
Huangshan	1.534	1.232 - 1.911	1.532	1.223 - 1.919	0.615	0.484 - 0.782	0.636	0.474 - 0.854	1.064	0.869 - 1.303	1.078	0.872 - 1.334	1.019	0.786 - 1.322	0.972	0.741 - 1.275
Luan	1.341	1.126 - 1.597	1.376	1.148 - 1.649	0.744	0.624 - 0.888	0.855	0.689 - 1.061	1.011	0.853 - 1.198	0.964	0.809 - 1.149	0.687	0.561 - 0.841	0.679	0.546 - 0.845
Maanshan	1.194	1.003 - 1.421	1.160	0.963 - 1.397	0.900	0.734 - 1.102	0.845	0.650 - 1.100	0.988	0.807 - 1.208	1.006	0.818 - 1.237	0.808	0.666 - 0.980	0.835	0.673 - 1.035
Suzhou	1.113	0.937 - 1.322	1.084	0.899 - 1.308	0.977	0.814 - 1.173	1.057	0.845 - 1.324	0.923	0.759 - 1.124	0.881	0.708 - 1.095	0.634	0.520 - 0.772	0.652	0.530 - 0.801
Tongling	1.497	1.205 - 1.859	1.431	1.135 - 1.804	0.975	0.747 - 1.271	0.930	0.666 - 1.299	1.020	0.782 - 1.329	1.024	0.782 - 1.341	0.912	0.716 - 1.162	0.958	0.738 - 1.243
Wuhu	1.021	0.836 - 1.247	1.052	0.857 - 1.292	0.738	0.571 - 0.953	0.653	0.468 - 0.911	0.864	0.673 - 1.109	0.884	0.686 - 1.140	0.710	0.573 - 0.881	0.689	0.549 - 0.866
Xuancheng	1.262	1.043 - 1.526	1.271	1.046 - 1.545	0.749	0.581 - 0.965	0.819	0.599 - 1.119	0.995	0.783 - 1.263	1.001	0.787 - 1.274	0.927	0.753 - 1.142	0.947	0.761 - 1.179
Meta-results	1.327	1.244 - 1.415	1.309	1.229 - 1.394	0.852	0.794 - 0.914	0.857	0.785 - 0.936	1.014	0.966 - 1.066	1.005	0.954 - 1.060	0.897	0.823 - 0.976	0.926	0.837 - 1.024

TB: tuberculosis; RR: relative risk; CI: 95% confidence intervals.

* Unadjusted model; ** The model adjusted for $PM_{2.5}$ and O_3.
