# Supplementary material for: Ambient temperature variability and newly diagnosed tuberculosis: Evidence from a time-stratified case-crossover study
Source: Environ Epidemiol. 2026 Oct 6;10(5):e531. doi: 10.1097/EE9.0000000000000531 (PMC13645473; doi:10.1097/EE9.0000000000000531)
Supplement: Supplementary file 6 [file ee9-10-e531-s006.pdf]

Supplementary Table 4. Season-stratified analysis of the associations between extreme temperature exposure and newly diagnosed TB

City	Extremely temperature (Cold season)								Extremely temperature (Warm season)							
	Heat				Cold				Heat				Cold			
	RR*	95% CI	aRR**	95% CI	RR*	95% CI	aRR**	95% CI	RR*	95% CI	aRR**	95% CI	RR*	95% CI	aRR**	95% CI
Anqing	0.997	0.856 - 1.162	0.956	0.807 - 1.131	0.927	0.807 - 1.066	0.961	0.830 - 1.112	1.073	0.906 - 1.273	1.216	1.013 - 1.460	0.996	0.864 - 1.147	0.947	0.808 - 1.110
Bengbu	0.935	0.779 - 1.122	0.933	0.748 - 1.165	0.875	0.751 - 1.021	0.886	0.751 - 1.046	1.085	0.875 - 1.346	1.169	0.918 - 1.489	1.037	0.861 - 1.249	0.979	0.801 - 1.197
Bozhou	1.039	0.889 - 1.215	1.063	0.892 - 1.267	0.896	0.779 - 1.032	0.855	0.737 - 0.992	1.115	0.917 - 1.356	1.289	1.032 - 1.611	1.046	0.901 - 1.215	0.948	0.809 - 1.111
Chizhou	0.894	0.727 - 1.098	0.872	0.692 - 1.099	0.945	0.783 - 1.141	0.931	0.767 - 1.131	1.513	1.163 - 1.968	1.501	1.128 - 1.998	1.020	0.805 - 1.293	0.967	0.738 - 1.267
Chuzhou	1.055	0.892 - 1.248	1.096	0.899 - 1.335	0.934	0.811 - 1.074	0.945	0.815 - 1.096	1.320	1.071 - 1.627	1.323	1.056 - 1.657	0.942	0.779 - 1.140	0.91	0.739 - 1.120
Fuyang	1.106	0.949 - 1.289	1.049	0.869 - 1.266	1.043	0.916 - 1.118	1.041	0.904 - 1.197	1.118	0.946 - 1.321	1.127	0.930 - 1.365	1.148	0.999 - 1.320	1.157	0.996 - 1.345
Hefei	1.038	0.914 - 1.179	1.042	0.898 - 1.210	0.942	0.844 - 1.051	0.965	0.859 - 1.084	1.218	1.048 - 1.415	1.271	1.079 - 1.498	0.983	0.862 - 1.122	0.933	0.808 - 1.078
Huaibei	0.892	0.726 - 1.096	0.846	0.656 - 1.093	0.793	0.672 - 0.937	0.799	0.665 - 0.959	0.779	0.625 - 0.971	0.793	0.616 - 1.021	0.988	0.835 - 1.170	0.954	0.797 - 1.141
Huainan	0.999	0.823 - 1.213	1.024	0.810 - 1.295	1.008	0.854 - 1.191	0.973	0.816 - 1.160	1.206	0.951 - 1.530	1.255	0.966 - 1.631	0.951	0.787 - 1.150	0.957	0.780 - 1.174
Huangshan	1.252	1.004 - 1.562	1.200	0.952 - 1.513	1.060	0.871 - 1.289	1.085	0.890 - 1.322	1.014	0.810 - 1.270	0.977	0.766 - 1.245	1.119	0.886 - 1.413	1.198	0.912 - 1.574
Luan	1.027	0.876 - 1.202	0.994	0.830 - 1.190	0.793	0.688 - 0.913	0.773	0.667 - 0.895	1.154	0.949 - 1.404	1.163	0.943 - 1.436	1.098	0.934 - 1.291	1.005	0.838 - 1.204
Maanshan	1.073	0.895 - 1.286	1.047	0.843 - 1.300	0.979	0.837 - 1.146	1.014	0.856 - 1.199	1.484	1.189 - 1.852	1.491	1.174 - 1.894	0.988	0.800 - 1.222	0.965	0.766 - 1.216
Suzhou	0.947	0.798 - 1.123	0.939	0.770 - 1.144	0.854	0.744 - 0.981	0.861	0.743 - 0.997	1.081	0.896 - 1.304	1.025	0.833 - 1.261	1.061	0.912 - 1.235	1.075	0.914 - 1.264
Tongling	0.928	0.731 - 1.179	0.850	0.652 - 1.107	1.184	0.962 - 1.458	1.277	1.030 - 1.583	1.075	0.817 - 1.413	1.056	0.788 - 1.415	0.969	0.747 - 1.258	1.156	0.864 - 1.548
Wuhu	1.207	0.980 - 1.488	1.040	0.820 - 1.319	0.831	0.692 - 0.998	0.888	0.735 - 1.072	1.349	1.040 - 1.749	1.334	1.009 - 1.764	1.119	0.861 - 1.454	1.002	0.754 - 1.334
Xuancheng	1.262	1.026 - 1.553	1.225	0.979 - 1.534	0.856	0.710 - 1.032	0.892	0.736 - 1.082	1.201	0.950 - 1.517	1.187	0.928 - 1.518	0.845	0.670 - 1.067	0.748	0.573 - 0.978
Meta-results	1.204	1.059 - 1.369	1.231	1.078 - 1.405	0.809	0.725 - 0.903	0.779	0.692 - 0.876	1.493	1.324 - 1.684	1.449	1.278 - 1.644	1.100	0.947 - 1.278	1.102	0.942 - 1.289

TB: tuberculosis; RR: relative risk; CI: 95% confidence intervals.

* Unadjusted model; ** The model adjusted for $PM_{2.5}$ and O_3.
