# Supplementary material for: Ambient temperature variability and newly diagnosed tuberculosis: Evidence from a time-stratified case-crossover study
Source: Environ Epidemiol. 2026 Oct 6;10(5):e531. doi: 10.1097/EE9.0000000000000531 (PMC13645473; doi:10.1097/EE9.0000000000000531)
Supplement: Supplementary file 7 [file ee9-10-e531-s007.pdf]

Supplementary Table 5. Region-stratified analysis of the associations between extreme temperature exposure and newly diagnosed TB

City	Extremely temperature							
	Heat				Cold			
	RR*	95% CI	aRR**	95% CI	RR*	95% CI	aRR**	95% CI
**North**								
Bengbu	1.162	1.010 - 1.337	1.271	1.098 - 1.472	1.050	0.913 - 1.209	0.916	0.778 - 1.078
Bozhou	1.123	0.986 - 1.278	1.122	0.973 - 1.294	0.873	0.766 - 0.996	0.905	0.780 - 1.051
Fuyang	1.069	0.961 - 1.189	1.102	0.983 - 1.235	0.975	0.876 - 1.085	0.901	0.796 - 1.020
Huaibei	1.093	0.949 - 1.258	1.062	0.915 - 1.234	0.849	0.734 - 0.982	0.865	0.727 - 1.029
Huainan	1.051	0.918 - 1.202	1.074	0.931 - 1.239	0.821	0.712 - 0.946	0.761	0.644 - 0.898
Suzhou	0.979	0.842 - 1.139	1.070	0.910 - 1.259	0.832	0.714 - 0.968	0.705	0.599 - 0.831
Meta-results	1.088	1.026 - 1.154	1.125	1.054 - 1.202	0.938	0.862 - 1.020	0.875	0.793 - 0.965
**South**								
Anqing	1.175	1.046 - 1.320	1.230	1.093 - 1.384	0.944	0.837 - 1.063	0.850	0.745 - 0.970
Hefei	1.155	1.052 - 1.269	1.193	1.082 - 1.315	0.816	0.737 - 0.903	0.764	0.681 - 0.857
Huangshan	1.237	1.061 - 1.443	1.252	1.070 - 1.465	0.837	0.700 - 1.000	0.742	0.614 - 0.898
Luan	1.180	1.035 - 1.346	1.228	1.073 - 1.405	0.736	0.639 - 0.849	0.665	0.568 - 0.778
Maanshan	1.060	0.924 - 1.216	1.105	0.959 - 1.274	0.856	0.741 - 0.990	0.750	0.633 - 0.888
Chizhou	1.190	1.018 - 1.391	1.205	1.027 - 1.413	0.980	0.831 - 1.154	0.942	0.780 - 1.137
Chuzhou	1.128	0.988 - 1.288	1.273	1.109 - 1.413	0.927	0.806 - 1.066	0.761	0.655 - 0.884
Tongling	1.217	1.028 - 1.441	1.278	1.073 - 1.521	0.936	0.782 - 1.121	0.837	0.685 - 1.024
Wuhu	0.926	0.789 - 1.087	1.003	0.852 - 1.179	0.750	0.636 - 0.884	0.620	0.518 - 0.743
Xuancheng	1.094	0.944 - 1.269	1.121	0.965 - 1.303	0.843	0.719 - 0.989	0.835	0.702 - 0.993
Meta-results	1.164	1.100 - 1.231	1.215	1.155 - 1.278	0.894	0.833 - 0.960	0.804	0.742 - 0.871

TB: tuberculosis; RR: relative risk; CI: 95% confidence intervals.

* Unadjusted model; ** The model adjusted for $PM_{2.5}$ and O_3.
