# Supplementary material for: Ambient temperature variability and newly diagnosed tuberculosis: Evidence from a time-stratified case-crossover study
Source: Environ Epidemiol. 2026 Oct 6;10(5):e531. doi: 10.1097/EE9.0000000000000531 (PMC13645473; doi:10.1097/EE9.0000000000000531)
Supplement: Supplementary file 8 [file ee9-10-e531-s008.pdf]

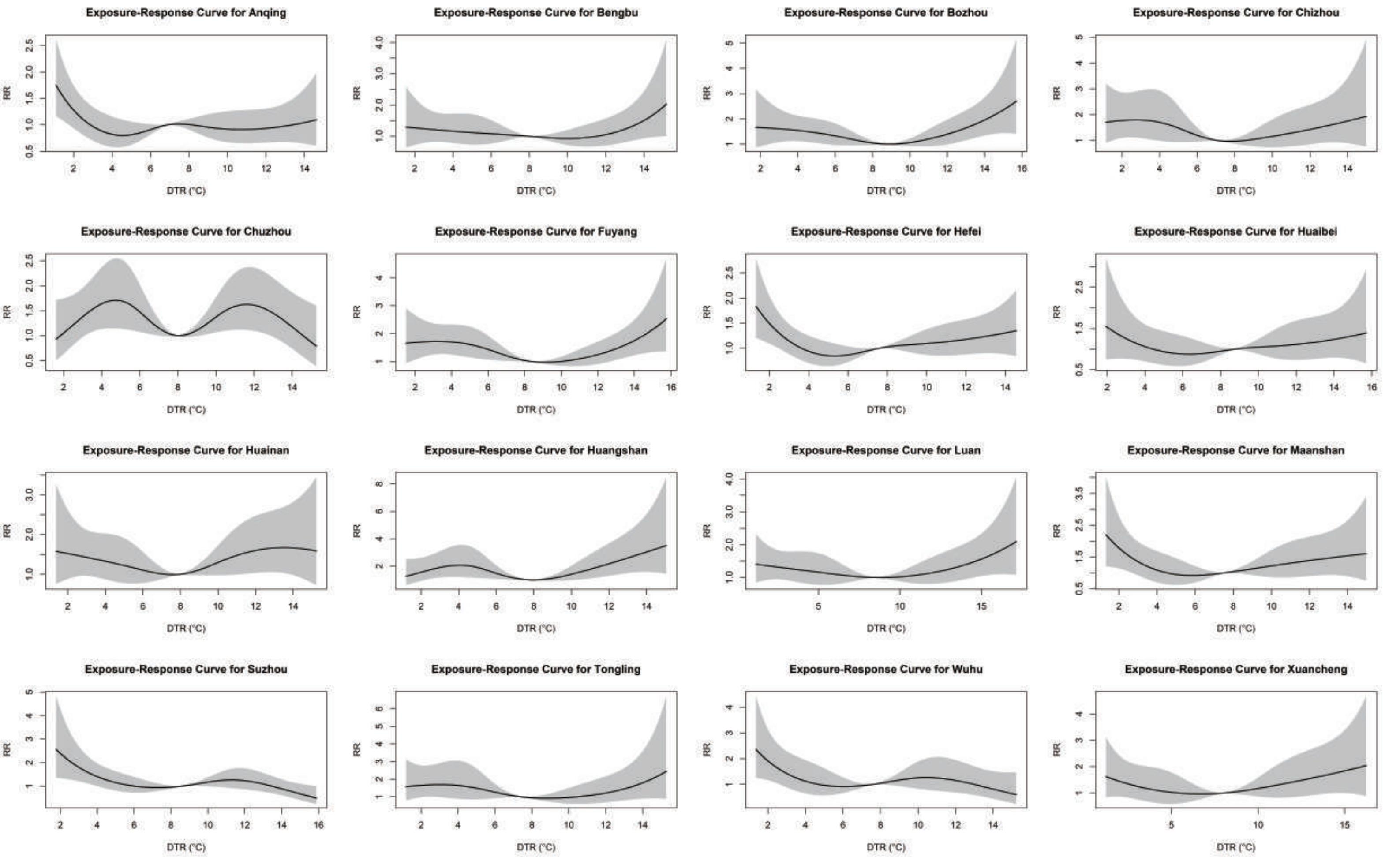
Exposure-Response Curve for Anqing
RR
DTR (°C)
Exposure-Response Curve for Bengbu
RR
DTR (°C)
Exposure-Response Curve for Bozhou
RR
DTR (°C)
Exposure-Response Curve for Chizhou
RR
DTR (°C)
Exposure-Response Curve for Chuzhou
RR
DTR (°C)
Exposure-Response Curve for Fuyang
RR
DTR (°C)
Exposure-Response Curve for Hefei
RR
DTR (°C)
Exposure-Response Curve for Huaibei
RR
DTR (°C)
Exposure-Response Curve for Huainan
RR
DTR (°C)
Exposure-Response Curve for Huangshan
RR
DTR (°C)
Exposure-Response Curve for Luan
RR
DTR (°C)
Exposure-Response Curve for Maanshan
RR
DTR (°C)
Exposure-Response Curve for Suzhou
RR
DTR (°C)
Exposure-Response Curve for Tongling
RR
DTR (°C)
Exposure-Response Curve for Wuhu
RR
DTR (°C)
Exposure-Response Curve for Xuancheng
RR
DTR (°C)
