# Supplementary material for: Ambient temperature variability and newly diagnosed tuberculosis: Evidence from a time-stratified case-crossover study
Source: Environ Epidemiol. 2026 Oct 6;10(5):e531. doi: 10.1097/EE9.0000000000000531 (PMC13645473; doi:10.1097/EE9.0000000000000531)
Supplement: Supplementary file 9 [file ee9-10-e531-s009.pdf]

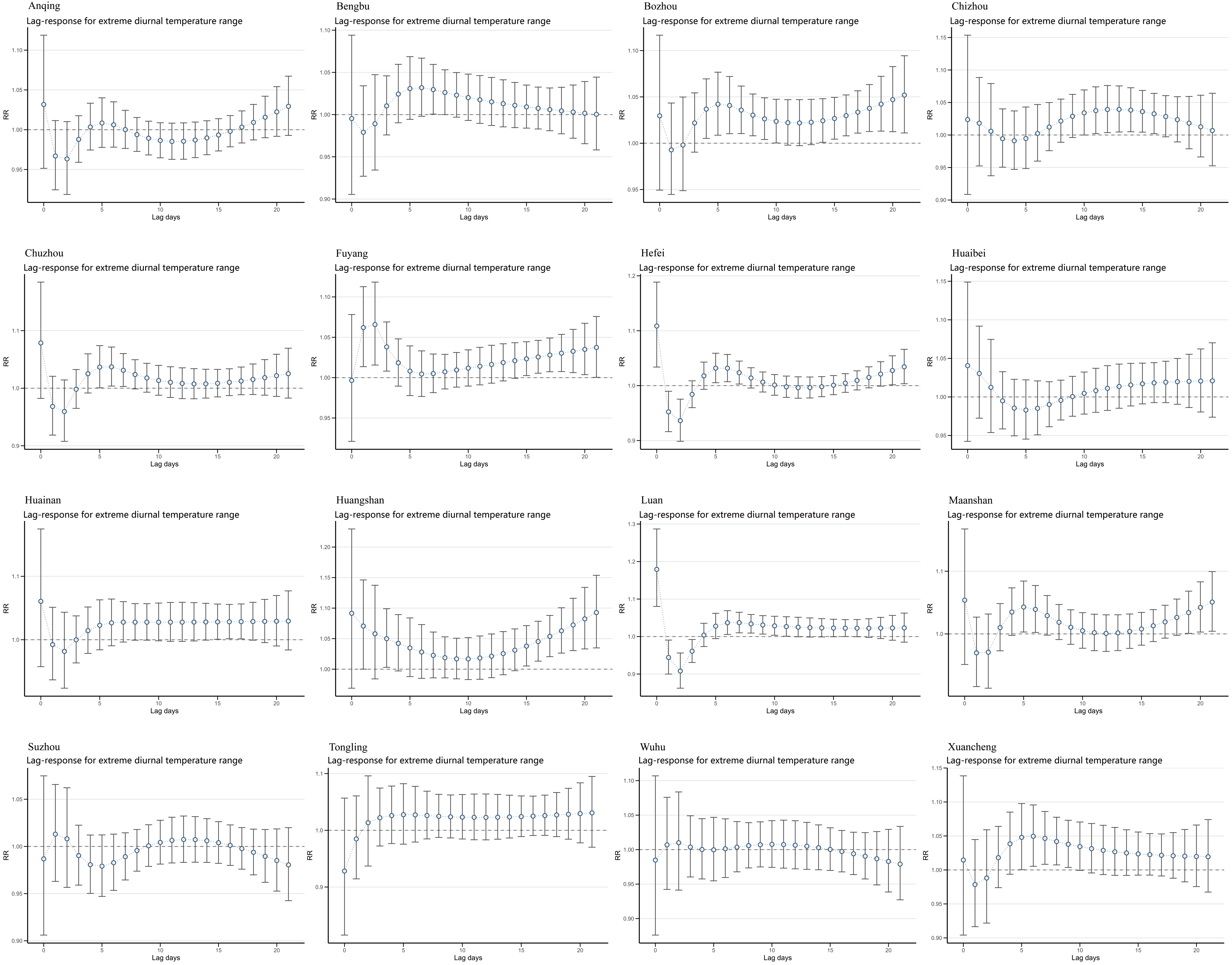

Anqing
Lag-response for extreme diurnal temperature range
Bengbu
Lag-response for extreme diurnal temperature range
Bozhou
Lag-response for extreme diurnal temperature range
Chizhou
Lag-response for extreme diurnal temperature range
Chuzhou
Lag-response for extreme diurnal temperature range
Fuyang
Lag-response for extreme diurnal temperature range
Hefei
Lag-response for extreme diurnal temperature range
Huaibei
Lag-response for extreme diurnal temperature range
Huainan
Lag-response for extreme diurnal temperature range
Huangshan
Lag-response for extreme diurnal temperature range
Luan
Lag-response for extreme diurnal temperature range
Maanshan
Lag-response for extreme diurnal temperature range
Suzhou
Lag-response for extreme diurnal temperature range
Tongling
Lag-response for extreme diurnal temperature range
Wuhu
Lag-response for extreme diurnal temperature range
Xuancheng
Lag-response for extreme diurnal temperature range
RR
Lag days
