# Supplementary material for: Ambient temperature variability and newly diagnosed tuberculosis: Evidence from a time-stratified case-crossover study
Source: Environ Epidemiol. 2026 Oct 6;10(5):e531. doi: 10.1097/EE9.0000000000000531 (PMC13645473; doi:10.1097/EE9.0000000000000531)
Supplement: Supplementary file 10 [file ee9-10-e531-s010.pdf]

**Supplementary Table 6. Subgroup analyses of the associations between DTR and newly diagnosed TB by sex and age**

| City | DTR | | | | | |
|---|---|---|---|---|---|---|
| | **Male** | **Female** | ***P* for heterogeneity** | **Age<60** | **Age⩾60** | ***P* for heterogeneity** |
| Anqing | 0.922 (0.632-1.345) | 1.051 (0.615-1.798) | 0.696 | 0.940 (0.611-1.446) | 0.976 (0.631-1.511) | 0.904 |
| Bengbu | 1.203 (0.761-1.901) | 1.479 (0.778-2.812) | 0.608 | 1.190 (0.737-1.922) | 1.442 (0.814-2.555) | 0.614 |
| Bozhou | 1.894 (1.284-2.794) | 1.839 (1.121-3.019) | 0.927 | 1.447 (0.948-2.209) | 2.432 (1.571-3.764) | 0.094 |
| Chizhou | 1.777 (1.006-3.139) | 1.037 (0.475-2.264) | 0.275 | 1.063 (0.573-1.971) | 2.244 (1.163-4.328) | 0.104 |
| Chuzhou | 1.560 (1.009-2.413) | 1.028 (0.572-1.845) | 0.263 | 1.229 (0.765-1.972) | 1.590 (0.959-2.637) | 0.466 |
| Fuyang | 1.594 (1.113-2.283) | 1.842 (1.154-2.940) | 0.631 | 1.490 (1.018-2.181) | 1.910 (1.263-2.889) | 0.387 |
| Hefei | 1.260 (0.907-1.750) | 1.137 (0.747-1.729) | 0.706 | 1.186 (0.843-1.668) | 1.284 (0.867-1.900) | 0.765 |
| Huaibei | 1.253 (0.762-2.062) | 1.148 (0.600-2.197) | 0.834 | 1.639 (0.997-2.694) | 0.726 (0.382-1.381) | 0.049 |
| Huainan | 1.767 (1.080-2.891) | 1.445 (0.778-2.682) | 0.618 | 1.858 (1.118-3.088) | 1.460 (0.828-2.577) | 0.535 |
| Huangshan | 2.333 (1.262-4.315) | 3.445 (1.479-8.024) | 0.465 | 1.991 (1.006-3.940) | 3.581 (1.736-7.386) | 0.248 |
| Luan | 1.504 (0.977-2.317) | 1.593 (0.922-2.753) | 0.872 | 1.806 (1.148-2.841) | 1.264 (0.768-2.078) | 0.299 |
| Maanshan | 1.435 (0.865-2.382) | 1.544 (0.783-3.043) | 0.865 | 1.746 (0.996-3.062) | 1.154 (0.653-2.040) | 0.310 |
| Suzhou | 1.093 (0.735-1.627) | 0.589 (0.348-0.994) | 0.066 | 0.819 (0.540-1.242) | 1.095 (0.681-1.761) | 0.368 |
| Tongling | 1.457 (0.757-2.804) | 1.546 (0.657-3.638) | 0.914 | 1.371 (0.677-2.777) | 1.603 (0.767-3.348) | 0.764 |
| Wuhu | 0.949 (0.539-1.671) | 1.074 (0.529-2.178) | 0.789 | 0.905 (0.496-1.650) | 1.126 (0.592-2.142) | 0.627 |
| Xuancheng | 1.372 (0.766-2.459) | 3.170 (1.461-6.876) | 0.090 | 1.728 (0.910-3.281) | 1.712 (0.895-3.276) | 0.984 |
| Meta-results | 1.351 (1.187-1.538) | 1.304 (1.077-1.578) | 0.764 | 1.279 (1.107-1.478) | 1.401 (1.173-1.673) | 0.435 |

DTR: diurnal temperature range; TB: tuberculosis; RR: relative risk; CI: 95% confidence intervals.
