# Supplementary material for: Ambient temperature variability and newly diagnosed tuberculosis: Evidence from a time-stratified case-crossover study
Source: Environ Epidemiol. 2026 Oct 6;10(5):e531. doi: 10.1097/EE9.0000000000000531 (PMC13645473; doi:10.1097/EE9.0000000000000531)
Supplement: Supplementary file 11 [file ee9-10-e531-s011.pdf]

Supplementary Table 7. Stratified analysis of the associations between DTR and newly diagnosed TB before and after the COVID-19 pandemic

City	Pre-pandemic period				Post-pandemic period			
	DTR							
	RR*	95% CI	aRR**	95% CI	RR*	95% CI	aRR**	95% CI
Anqing	1.113	0.685 - 1.809	1.072	0.658 - 1.746	0.776	0.472 - 1.275	0.779	0.473 - 1.282
Bengbu	1.355	0.765 - 2.398	1.281	0.722 - 2.271	1.009	0.535 - 1.902	1.029	0.545 - 1.943
Bozhou	2.394	1.462 - 3.923	2.330	1.416 - 3.833	1.179	0.646 - 2.153	1.119	0.610 - 2.051
Chizhou	1.870	0.883 - 3.959	1.834	0.865 - 3.889	1.346	0.637 - 2.843	1.366	0.646 - 2.888
Chuzhou	1.356	0.812 - 2.266	1.456	0.865 - 2.451	1.196	0.594 - 2.408	1.210	0.600 - 2.439
Fuyang	1.578	1.000 - 2.490	1.670	1.052 - 2.652	1.651	0.970 - 2.813	1.642	0.961 - 2.806
Hefei	1.149	0.770 - 1.714	1.152	0.771 - 1.723	1.307	0.824 - 2.073	1.315	0.828 - 2.089
Huaibei	1.163	0.644 - 2.102	1.143	0.632 - 2.067	1.158	0.598 - 2.243	1.067	0.551 - 2.065
Huainan	1.428	0.777 - 2.624	1.433	0.778 - 2.641	1.823	0.906 - 3.667	1.884	0.934 - 3.799
Huangshan	4.046	1.869 - 8.758	4.011	1.832 - 8.780	1.428	0.632 - 3.225	1.406	0.622 - 3.178
Luan	1.665	0.995 - 2.786	1.629	0.971 - 2.731	1.084	0.580 - 2.025	1.081	0.578 - 2.022
Maanshan	1.211	0.693 - 2.117	1.307	0.742 - 2.301	1.645	0.773 - 3.499	1.650	0.775 - 3.514
Suzhou	1.031	0.636 - 1.672	1.045	0.644 - 1.697	0.839	0.462 - 1.526	0.805	0.442 - 1.467
Tongling	1.836	0.858 - 3.931	1.829	0.851 - 3.930	1.379	0.502 - 3.790	1.247	0.452 - 3.441
Wuhu	1.403	0.687 - 2.863	1.315	0.640 - 2.700	0.630	0.274 - 1.445	0.612	0.266 - 1.406
Xuancheng	1.671	0.816 - 3.424	1.728	0.840 - 3.554	2.080	0.881 - 4.908	2.159	0.915 - 5.094
Meta-results	1.385	1.166 - 1.645	1.389	1.166 - 1.654	1.150	0.960 - 1.377	1.148	0.954 - 1.380

DTR: diurnal temperature range; TB: tuberculosis; RR: relative risk; CI: 95% confidence intervals.

* Unadjusted model; ** The model adjusted for $PM_{2.5}$ and O_3.
