# Supplementary material for: Ambient temperature variability and newly diagnosed tuberculosis: Evidence from a time-stratified case-crossover study
Source: Environ Epidemiol. 2026 Oct 6;10(5):e531. doi: 10.1097/EE9.0000000000000531 (PMC13645473; doi:10.1097/EE9.0000000000000531)
Supplement: Supplementary file 12 [file ee9-10-e531-s012.pdf]

Supplementary Table 8. Season-stratified analysis of the associations between DTR and newly diagnosed TB

City	Extremely temperature (Cold season)				Extremely temperature (Warm season)			
	DTR				DTR			
	RR*	95% CI	aRR**	95% CI	RR*	95% CI	aRR**	95% CI
Anqing	1.327	0.805 - 2.188	1.345	0.808 - 2.239	0.899	0.580 - 1.393	0.864	0.553 - 1.351
Bengbu	1.039	0.575 - 1.877	1.225	0.667 - 2.250	1.074	0.633 - 1.822	1.105	0.648 - 1.886
Bozhou	1.515	0.911 - 2.519	1.754	1.041 - 2.955	1.536	0.934 - 2.526	1.455	0.877 - 2.416
Chizhou	1.883	0.902 - 3.933	2.123	0.999 - 4.514	1.558	0.770 - 3.152	1.696	0.828 - 3.472
Chuzhou	1.258	0.778 - 2.035	1.302	0.798 - 2.125	1.637	0.990 - 2.707	1.700	1.012 - 2.854
Fuyang	1.958	1.204 - 3.184	1.977	1.196 - 3.270	1.410	0.897 - 2.214	1.443	0.910 - 2.289
Hefei	1.507	1.033 - 2.198	1.568	1.063 - 2.314	0.983	0.664 - 1.454	1.036	0.697 - 1.541
Huaibei	1.485	0.786 - 2.803	1.597	0.827 - 3.085	0.934	0.534 - 1.631	0.843	0.479 - 1.484
Huainan	1.253	0.697 - 2.255	1.253	0.686 - 2.286	1.350	0.762 - 2.395	1.411	0.789 - 2.524
Huangshan	1.300	0.587 - 2.882	1.374	0.613 - 3.079	2.024	1.000 - 4.096	1.779	0.858 - 3.688
Luan	1.311	0.823 - 2.087	1.317	0.810 - 2.140	1.021	0.616 - 1.693	1.002	0.599 - 1.674
Maanshan	1.310	0.770 - 2.228	1.244	0.723 - 2.140	1.490	0.866 - 2.566	1.534	0.880 - 2.676
Suzhou	3.519	2.049 - 6.043	4.098	2.348 - 7.154	0.940	0.600 - 1.472	0.898	0.567 - 1.421
Tongling	1.819	0.865 - 3.826	1.806	0.846 - 3.855	0.578	0.254 - 1.317	0.602	0.262 - 1.381
Wuhu	0.855	0.460 - 1.589	0.847	0.451 - 1.589	1.933	0.963 - 3.881	1.649	0.810 - 3.356
Xuancheng	1.235	0.606 - 2.517	1.085	0.527 - 2.233	2.115	1.060 - 4.221	2.221	1.100 - 4.485
Meta-results	1.438	1.220 - 1.695	1.479	1.226 - 1.783	1.222	1.045 - 1.429	1.214	1.032 - 1.429

DTR: diurnal temperature range; TB: tuberculosis; RR: relative risk; CI: 95% confidence intervals.

* Unadjusted model; ** The model adjusted for $PM_{2.5}$ and O_3.
