# Supplementary material for: Ambient temperature variability and newly diagnosed tuberculosis: Evidence from a time-stratified case-crossover study
Source: Environ Epidemiol. 2026 Oct 6;10(5):e531. doi: 10.1097/EE9.0000000000000531 (PMC13645473; doi:10.1097/EE9.0000000000000531)
Supplement: Supplementary file 13 [file ee9-10-e531-s013.pdf]

Supplementary Table 9. Region-stratified analysis of the associations between DTR and newly diagnosed TB

City	DTR			
	**RR***	**95% CI**	**aRR****	**95% CI**
**North**				
Bengbu	1.278	0.837 - 1.950	1.242	0.814 - 1.896
Bozhou	1.871	1.299 - 2.695	1.793	1.241 - 2.591
Fuyang	1.663	1.185 - 2.333	1.708	1.212 - 2.406
Huaibei	1.223	0.794 - 1.882	1.184	0.768 - 1.825
Huainan	1.668	1.057 - 2.633	1.669	1.057 - 2.633
Suzhou	0.922	0.638 - 1.334	0.914	0.632 - 1.322
Meta-results	1.362	1.102 - 1.683	1.338	1.077 - 1.664
**South**				
Anqing	0.955	0.675 - 1.351	0.935	0.661 - 1.324
Hefei	1.219	0.903 - 1.646	1.223	0.905 - 1.654
Huangshan	2.637	1.517 - 4.585	2.567	1.473 - 4.474
Luan	1.523	1.026 - 2.260	1.502	1.011 - 2.231
Maanshan	1.458	0.934 - 2.276	1.504	0.961 - 2.352
Chizhou	1.536	0.912 - 2.585	1.509	0.896 - 2.541
Chuzhou	1.387	0.926 - 2.078	1.431	0.952 - 2.150
Tongling	1.492	0.817 - 2.723	1.461	0.799 - 2.673
Wuhu	0.982	0.576 - 1.676	0.937	0.548 - 1.600
Xuancheng	1.718	0.993 - 2.972	1.769	1.022 - 3.062
Meta-results	1.319	1.111 - 1.566	1.312	1.100 - 1.564

DTR: diurnal temperature range; TB: tuberculosis; RR: relative risk; CI: 95% confidence intervals.

* Unadjusted model; ** The model adjusted for $PM_{2.5}$ and O_3.
