# Supplementary material for: Ambient temperature variability and newly diagnosed tuberculosis: Evidence from a time-stratified case-crossover study
Source: Environ Epidemiol. 2026 Oct 6;10(5):e531. doi: 10.1097/EE9.0000000000000531 (PMC13645473; doi:10.1097/EE9.0000000000000531)
Supplement: Supplementary file 14 [file ee9-10-e531-s014.pdf]

Supplementary Table 10. Sensitivity analyses of spline function nodes under different knot placements

City	Knots: 10th, 50th and 90th percentiles			Knots: 10th, 75th and 90th		
	**Heat**	**Cold**	**DTR**	**Heat**	**Cold**	**DTR**
Anqing	1.158 (1.033 - 1.298)	0.946 (0.841 - 1.066)	0.995 (0.700 - 1.415)	1.161 (1.036 - 1.301)	0.932 (0.831 - 1.045)	1.044 (0.719 - 1.515)
Bengbu	1.121 (0.978 - 1.285)	1.051 (0.914 - 1.208)	1.247 (0.821 - 1.894)	1.122 (0.978 - 1.287)	0.990 (0.867 - 1.129)	1.240 (0.801 - 1.919)
Bozhou	1.087 (0.956 - 1.234)	0.877 (0.770 - 0.999)	1.857 (1.293 - 2.666)	1.075 (0.945 - 1.223)	0.851 (0.751 - 0.966)	2.003 (1.382 - 2.903)
Chizhou	1.166 (1.001 - 1.358)	0.983 (0.835 - 1.156)	1.515 (0.889 - 2.583)	1.177 (1.009 - 1.372)	0.975 (0.834 - 1.141)	1.542 (0.879 - 2.704)
Chuzhou	1.105 (0.972 - 1.257)	0.923 (0.804 - 1.061)	1.444 (0.965 - 2.161)	1.108 (0.973 - 1.262)	0.901 (0.789 - 1.029)	1.544 (1.011 - 2.359)
Fuyang	1.052 (0.947 - 1.168)	0.975 (0.877 - 1.085)	1.655 (1.177 - 2.327)	1.054 (0.949 - 1.171)	0.958 (0.865 - 1.061)	1.710 (1.196 - 2.445)
Hefei	1.142 (1.043 - 1.251)	0.818 (0.740 - 0.904)	1.315 (0.975 - 1.774)	1.153 (1.050 - 1.265)	0.809 (0.734 - 0.891)	1.433 (1.051 - 1.954)
Huaibei	1.064 (0.927 - 1.221)	0.852 (0.737 - 0.984)	1.203 (0.786 - 1.840)	1.062 (0.925 - 1.220)	0.832 (0.724 - 0.955)	1.078 (0.693 - 1.678)
Huainan	1.036 (0.908 - 1.181)	0.828 (0.719 - 0.953)	1.676 (1.067 - 2.631)	1.052 (0.920 - 1.203)	0.822 (0.718 - 0.941)	1.738 (1.086 - 2.783)
Huangshan	1.228 (1.057 - 1.426)	0.837 (0.701 - 0.998)	2.967 (1.701 - 5.177)	1.217 (1.049 - 1.412)	0.815 (0.687 - 0.967)	3.772 (2.110 - 6.741)
Luan	1.150 (1.012 - 1.306)	0.742 (0.645 - 0.854)	1.521 (1.025 - 2.257)	1.176 (1.033 - 1.340)	0.741 (0.646 - 0.849)	1.544 (1.028 - 2.317)
Maanshan	1.040 (0.910 - 1.189)	0.854 (0.739 - 0.986)	1.527 (0.985 - 2.367)	1.034 (0.904 - 1.182)	0.821 (0.715 - 0.943)	1.496 (0.946 - 2.365)
Suzhou	0.936 (0.807 - 1.087)	0.835 (0.718 - 0.971)	0.903 (0.627 - 1.302)	0.925 (0.796 - 1.076)	0.800 (0.691 - 0.925)	0.909 (0.619 - 1.336)
Tongling	1.186 (1.006 - 1.399)	0.934 (0.782 - 1.117)	1.442 (0.788 - 2.639)	1.174 (0.996 - 1.383)	0.895 (0.754 - 1.063)	1.473 (0.780 - 2.781)
Wuhu	0.900 (0.769 - 1.053)	0.748 (0.635 - 0.880)	0.987 (0.581 - 1.678)	0.891 (0.762 - 1.042)	0.717 (0.612 - 0.840)	0.906 (0.523 - 1.568)
Xuancheng	1.067 (0.924 - 1.232)	0.838 (0.716 - 0.981)	1.713 (0.994 - 2.953)	1.061 (0.918 - 1.225)	0.806 (0.692 - 0.937)	1.636 (0.937 - 2.857)
Meta-results	1.157 (1.114 - 1.203)	0.910 (0.863 - 0.958)	1.346 (1.184 - 1.531)	1.177 (1.131 - 1.225)	0.902 (0.857 - 0.950)	1.382 (1.186 - 1.610)

DTR: diurnal temperature range.
