# Supplementary material for: Ambient temperature variability and newly diagnosed tuberculosis: Evidence from a time-stratified case-crossover study
Source: Environ Epidemiol. 2026 Oct 6;10(5):e531. doi: 10.1097/EE9.0000000000000531 (PMC13645473; doi:10.1097/EE9.0000000000000531)
Supplement: Supplementary file 15 [file ee9-10-e531-s015.pdf]

Supplementary Table 11. Sensitivity analyses of extreme temperature exposure and newly diagnosed TB across different lag periods

City	Extremely temperature (Lag = 0-14 days)								Extremely temperature (Lag = 0-28 days)							
	Heat				Cold				Heat				Cold			
	RR*	95% CI	aRR**	95% CI	RR*	95% CI	aRR**	95% CI	RR*	95% CI	aRR**	95% CI	RR*	95% CI	aRR**	95% CI
Anqing	1.116	0.998 - 1.248	1.160	1.036 - 1.299	0.934	0.834 - 1.048	0.848	0.746 - 0.964	1.186	1.050 - 1.339	1.245	1.101 - 1.408	0.952	0.841 - 1.079	0.857	0.748 - 0.982
Bengbu	1.126	0.984 - 1.289	1.225	1.064 - 1.410	1.045	0.913 - 1.197	0.906	0.771 - 1.063	1.175	1.014 - 1.360	1.309	1.123 - 1.526	1.128	0.974 - 1.305	0.965	0.818 - 1.140
Bozhou	1.061	0.938 - 1.201	1.046	0.913 - 1.198	0.846	0.745 - 0.961	0.891	0.770 - 1.031	1.140	0.995 - 1.308	1.146	0.986 - 1.332	0.930	0.810 - 1.067	0.958	0.821 - 1.117
Chizhou	1.134	0.977 - 1.317	1.140	0.979 - 1.327	0.923	0.789 - 1.080	0.908	0.755 - 1.091	1.163	0.987 - 1.371	1.177	0.996 - 1.390	0.953	0.803 - 1.132	0.917	0.755 - 1.114
Chuzhou	1.075	0.947 - 1.221	1.202	1.053 - 1.373	0.937	0.820 - 1.071	0.767	0.664 - 0.887	1.118	0.973 - 1.284	1.273	1.102 - 1.470	0.948	0.819 - 1.097	0.776	0.664 - 0.906
Fuyang	1.049	0.947 - 1.162	1.077	0.966 - 1.201	0.959	0.864 - 1.063	0.888	0.786 -1.003	1.060	0.948 - 1.186	1.104	0.979 - 1.244	1.011	0.904 - 1.131	0.926	0.815 - 1.051
Hefei	1.136	1.039 - 1.243	1.177	1.071 - 1.293	0.849	0.771 - 0.935	0.787	0.703 - 0.880	1.160	1.052 - 1.279	1.200	1.084 - 1.329	0.815	0.733 - 0.906	0.766	0.680 - 0.862
Huaibei	1.074	0.939 - 1.228	1.048	0.909 - 1.208	0.863	0.751 - 0.992	0.879	0.741 - 1.042	1.132	0.976 - 1.312	1.100	0.940 - 1.288	0.859	0.737 - 1.000	0.867	0.726 - 1.036
Huainan	1.061	0.933 - 1.206	1.083	0.945 - 1.241	0.831	0.726 - 0.952	0.769	0.653 - 0.904	1.058	0.919- 1.219	1.092	0.940 - 1.269	0.876	0.756 - 1.015	0.800	0.675 - 0.948
Huangshan	1.215	1.049 - 1.407	1.222	1.050 - 1.421	0.859	0.726 - 1.016	0.763	0.637 - 0.914	1.206	1.027 - 1.417	1.223	1.038 - 1.441	0.811	0.673 - 0.978	0.720	0.591 - 0.878
Luan	1.165	1.028 - 1.322	1.210	1.063 - 1.376	0.781	0.682 - 0.894	0.698	0.600 - 0.813	1.172	1.022 - 1.343	1.220	1.061 - 1.404	0.704	0.607 -0.817	0.639	0.544 - 0.751
Maanshan	1.048	0.919 - 1.195	1.094	0.955 - 1.254	0.882	0.767 - 1.013	0.767	0.650 - 0.904	1.048	0.908 - 1.210	1.108	0.955 - 1.285	0.873	0.751 - 1.016	0.752	0.633 - 0.894
Suzhou	0.983	0.851 - 1.137	1.089	0.933 - 1.272	0.886	0.766 - 1.025	0.742	0.633 - 0.869	0.996	0.849 - 1.168	1.088	0.917 - 1.292	0.862	0.735 - 1.011	0.729	0.615 - 0.865
Tongling	1.168	0.993 - 1.372	1.218	1.031 - 1.438	0.908	0.764 - 1.078	0.814	0.670 - 0.989	1.232	1.034 - 1.468	1.305	1.089 - 1.563	0.970	0.805 - 1.169	0.857	0.698 - 1.053
Wuhu	0.928	0.795 - 1.082	1.004	0.859 - 1.173	0.801	0.685 - 0.937	0.650	0.545 - 0.775	0.927	0.784 - 1.096	1.006	0.849 - 1.192	0.723	0.609 - 0.859	0.608	0.505 - 0.733
Xuancheng	1.042	0.904 - 1.200	1.063	0.921 - 1.228	0.853	0.733 - 0.992	0.843	0.713 - 0.997	1.067	0.913 - 1.247	1.092	0.932 - 1.279	0.830	0.703 - 0.981	0.825	0.690 - 0.987
Meta-results	1.131	1.094 - 1.168	1.173	1.134 - 1.214	0.915	0.879 - 0.952	0.833	0.791 - 0.876	1.182	1.137 - 1.229	1.235	1.184 - 1.288	0.933	0.873 - 0.997	0.846	0.782 - 0.915

TB: tuberculosis; RR: relative risk; CI: 95% confidence intervals.

* Unadjusted model; ** The model adjusted for PM2.5 and O3.
