# Supplementary material for: Ambient temperature variability and newly diagnosed tuberculosis: Evidence from a time-stratified case-crossover study
Source: Environ Epidemiol. 2026 Oct 6;10(5):e531. doi: 10.1097/EE9.0000000000000531 (PMC13645473; doi:10.1097/EE9.0000000000000531)
Supplement: Supplementary file 16 [file ee9-10-e531-s016.pdf]

Supplementary Table 12. Sensitivity analyses of DTR and newly diagnosed TB across different lag periods

City	DTR (Lag = 0-14 days)				DTR (Lag = 0-28 days)			
	RR*	95% CI	aRR**	95% CI	RR*	95% CI	aRR**	95% CI
Anqing	0.867	0.668 - 1.124	0.846	0.651 - 1.100	1.119	0.737 - 1.698	1.085	0.713 - 1.649
Bengbu	1.202	0.878 - 1.645	1.168	0.852 - 1.600	1.458	0.856 - 2.483	1.389	0.815 - 2.369
Bozhou	1.284	0.984 - 1.675	1.230	0.940 - 1.611	2.330	1.450 - 3.746	2.233	1.386 - 3.599
Chizhou	1.201	0.812 - 1.777	1.205	0.814 - 1.786	1.326	0.696 - 2.527	1.328	0.697 - 2.532
Chuzhou	1.238	0.917 - 1.671	1.245	0.919 - 1.685	1.785	1.065 - 2.993	1.768	1.053 - 2.970
Fuyang	1.193	0.923 - 1.543	1.213	0.935 - 1.572	1.631	1.072 - 2.483	1.691	1.105 - 2.587
Hefei	0.970	0.777 - 1.212	0.968	0.775 - 1.210	1.391	0.959 - 2.019	1.383	0.952 - 2.010
Huaibei	0.904	0.662 - 1.234	0.868	0.634 - 1.189	1.222	0.693 - 2.154	1.169	0.661 - 2.067
Huainan	1.328	0.949 - 1.857	1.354	0.967 - 1.896	2.168	1.228 - 3.828	2.080	1.177 - 3.676
Huangshan	1.334	0.889 - 2.002	1.309	0.869 - 1.970	2.771	1.389 - 5.528	2.607	1.304 - 5.213
Luan	1.161	0.867 - 1.554	1.150	0.859 - 1.541	2.191	1.352 - 3.548	2.139	1.318 - 3.471
Maanshan	1.060	0.760 - 1.477	1.078	0.771 - 1.506	1.340	0.765 - 2.347	1.355	0.773 - 2.376
Suzhou	0.938	0.717 - 1.227	0.946	0.722 - 1.238	1.347	0.838 - 2.166	1.279	0.795 - 2.056
Tongling	1.025	0.655 - 1.604	1.012	0.646 - 1.586	1.263	0.612 - 2.603	1.218	0.589 - 2.520
Wuhu	1.079	0.726 - 1.603	1.015	0.681 - 1.512	1.378	0.714 - 2.657	1.269	0.657 - 2.452
Xuancheng	1.333	0.889 - 1.999	1.372	0.913 - 2.062	1.760	0.906 - 3.419	1.834	0.943 - 3.565
Meta-results	1.098	1.005 - 1.200	1.086	0.991 - 1.190	1.504	1.296 - 1.745	1.472	1.259 - 1.722

DTR: diurnal temperature range; TB: tuberculosis; RR: relative risk; CI: 95% confidence intervals.

* Unadjusted model; ** The model adjusted for $PM_{2.5}$ and O_3.
